# Interferometric Evidence of Nonvolatile Anomalous Phase Shifts in Exchange-Spin-Split Josephson Supercurrent Diodes

**DOI:** 10.1021/acsnano.5c17979

**Published:** 2026-01-22

**Authors:** Kun-Rok Jeon, Jae-Keun Kim, Jiho Yoon, Jae-Chun Jeon, Hyeon Han, Audrey Cottet, Takis Kontos, Stuart S. P. Parkin

**Affiliations:** † Department of Physics, Chung-Ang University (CAU), 06974 Seoul, Republic of Korea; ‡ Max Planck Institute of Microstructure Physics, Weinberg 2, 06120 Halle (Saale), Germany; § Department of Materials Science and Engineering, Pohang University of Science and Technology (POSTECH), 37673 Pohang, Republic of Korea; ∥ Laboratoire de Physique de l’Ecole Normale Supérieure, ENS, Université PSL, CNRS, Sorbonne Université, Université Paris-Diderot,Sorbonne Paris Cité, 75005 Paris, France; ⊥ Laboratoire de Physique et d’Etude des Matériaux, ESPCI Paris, PSL University, CNRS, Sorbonne Université, 75005 Paris, France

**Keywords:** exchange-spin-split Josephson supercurrent diodes, interfacial magnetic ordering, Rashba spin−orbit interaction, nonvolatile anomalous phase shifts, superconducting quantum interferometry

## Abstract

The recent realization of zero-field, polarity-reversible supercurrent rectification in proximity-magnetized Rashba-type Pt Josephson junctions (JJs) enables the development of superconducting logic circuits and cryogenic memory applications. Here, we demonstrate a *nonvolatile* anomalous phase shift φ_0_ directly probed via superconducting quantum interferometry, providing phase-sensitive evidence of spontaneous time-reversal symmetry breaking in these Rashba-type systems. By replacing the Pt barrier with 5d or 4d element layers exhibiting different (para)­magnetic susceptibilities, spin–orbit coupling properties, and electronic band structures, we elucidate the role of proximity effects in governing zero-field diode behavior. Ta (W) JJs exhibit zero-field diode efficiencies of ∼17% (∼5%) at 2 K, which are slightly (significantly) lower than those of Pt JJs. Notably, the diode polarity in Ta and W JJs is reversed relative to that in Pt JJs. Combined with the large zero-field diode efficiency (∼15% at 2 K) observed in highly magnetic-susceptible Pd JJs, these results show that nonvolatile φ_0_ and, consequently, zero-field diode performance can be tuned through proximity engineering of interfacial magnetic ordering and Rashba spin–orbit interaction.

## Graphical abstract

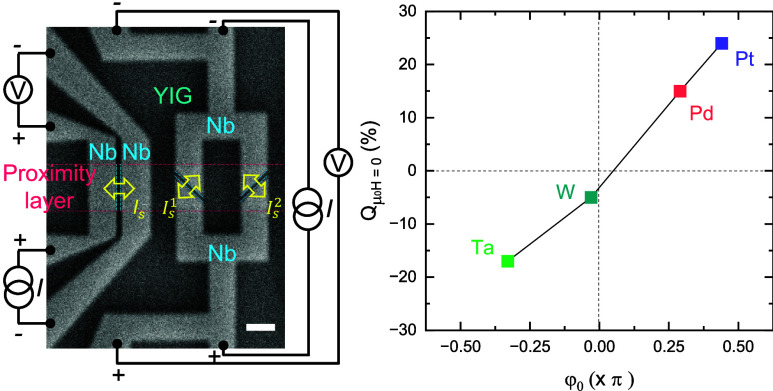

Spontaneous breaking of time-reversal symmetry in a noncentrosymmetric conductor leads to a nonreciprocal electronic transport^1–4^ at zero external magnetic field. This so-called zero-field and polarity-switchable rectification has been recently observed in the supercurrents of proximity-magnetized Rashba­(-type) Pt Josephson junctions (JJs),^5^ Co- or Fe-inserted noncentrosymmetric Nb/V/Ta artificial superlattices,^6,7^ gated small-twist-angle trilayer graphene,^8^ cross-like Nb planar JJs with an artificial vortex trap,^9^ and Nb/EuS proximity bilayers.^10^ The experimental realization of such zero-field polarity-switchable supercurrent rectification, especially in the form of lateral JJs,^5^ provides a technical progress for the development of ultralow-power rectifier circuits and nonvolatile (superconducting-)­phase memories.^11–13^

Zeeman-spin-splitting-driven Josephson supercurrent diodes^14–22^ with a nonmagnetic inversion-asymmetric barrier necessarily require an external magnetic field μ_0_ *H* to break the time-reversal symmetry. This applied μ_0_ *H* induces the nonzero ground-state anomalous phase shift φ_0_ ≠ 0 ^20–22^ in the current-phase relationship (CPR),^23^ resulting in a nonsinusoidal CPR characterized by *I*_*s*_(φ) ≠ −*I*_s_(−φ), where φ is the Josephson phase. This nonsinusoidal CPR, in conjunction with higher-order harmonics, leads to Josephson supercurrent nonreciprocity,^12–22^ manifesting as unequal positive and negative critical currents *I*_c_^+^ ≠ |*I*_c_^–^|. Here, φ_0_ scales with spin-splitting fields, the degree of inversion asymmetry and spin–orbit coupling (SOC),^17–22^ and the underlying CPR is approximately described as *I*_s_(φ) ≈ *I*_*c*1_ sin­(φ + φ_0_) + *I*_*c*2_ sin­(2φ). For a small second-harmonic contribution and small φ_0_, $Q=\frac{I_{c}^{+}-\left|I_{c}^{-}\right|}{I_{c}^{+}+\left|I_{c}^{-}\right|}\approx -\frac{I_{c2}\varphi_{0}}{I_{c1}}$ (see the Supporting Information for details). Note that the combination of φ_0_ and *I*_c2_ **sin­(2φ) leads to an anisotropic μ_0_ *H*-dependent *I*_c_ ^20–23^ for the Zeeman-spin-splitting-driven Josephson supercurrent diodes.^14–22^ On the other hand, exchange-spin-splitting-driven Josephson diodes^24–26^ with a magnetic inversion-asymmetric barrier, like the proximity-magnetized Pt JJs^5^ with Rashba SOC,^27^ can inherently possess φ_0_ even in the absence of μ_0_ *H*. This results in the nonvolatility of φ_0_ for μ_0_ *H* = 0, which, in turn, enables the spontaneous magnetization of φ_0_-barrier to induce the zero-field anisotropic *I*_c_ ^5,24–26^ and to control the polarization of supercurrent nonreciprocity by preconfiguring its remanent state. These exchange-spin-splitting-driven Josephson diodes^5,24–26^ are thus highly promising for future superconducting-phase memory and logic circuit applications.^11–13^ For instance, instead of using a φ (= 0 or π) junction^13^ as a memory cell for the Josephson phase memory, where the necessary arbitrary phase shift φ in the ground state is obtained by geometrically combining 0 and π JJs in parallel, the exchange-spin-split Josephson φ_0_-junctions having 0 < φ_0_ < π even in the form of a single junction can be utilized.

So far, unlike the case of the former Zeeman-spin-split Josephson φ_0_-junctions,^17–20^ the interferometry characterization^17–20^ of φ_0_ at zero magnetic field in the latter exchange-spin-split Josephson φ_0_-junctions and how this *nonvolatile* φ_0_ is linked to the zero-field nonreciprocal supercurrents remain to be investigated. In addition, a systematic study of how the choice of a proximity layer for the Josephson φ_0_-barrier affects the sign and magnitude of the resulting supercurrent nonreciprocity is outstanding not only for disentangling the intrinsic origin of zero-field supercurrent nonreciprocity from other extrinsic factors (e.g., spatially nonuniform supercurrents and rectifying motion of Josephson or Abrikosov vortices)^9,10^ but also for further improving/tuning the Josephson diode performance based on the more fundamental quantity of φ_0_ rather than the Josephson current–voltage characteristics.

In this work, by substituting the Pt Josephson φ_0_-barrier^5^ for either 5d or 4d element proximity layer (Figures 1A and S1) with different (para-)­magnetic susceptibility, SOC and electronic band structure, and fabricating a superconducting quantum interference device (SQUID) consisting of two symmetric Josephson φ_0_-junctions (Figure 1B), we are able to elucidate the role of interface magnetic ordering and Rashba effect in determining the zero-field Josephson diode properties, and to prove the existence of the nonvolatile φ_0_ and its correlation with the zero-field diode effect. In our experimental setup, a pair of Josephson φ_0_-junction and SQUID are fabricated on the same proximity layer (Figure 1C), which is (proximity-)­magnetized by a ferrimagnetic insulator Y_3_Fe_5_O_12_ (YIG) underneath it, allowing for a direct comparison between the nonreciprocal Josephson supercurrents and phase-resolved interferometer data. Note also that our SQUID geometry is conceived to detect both nonvolatile φ_0_ and the zero-field diode effect even in a single SQUID (Figure S2), as discussed below.

**Figure 1:**
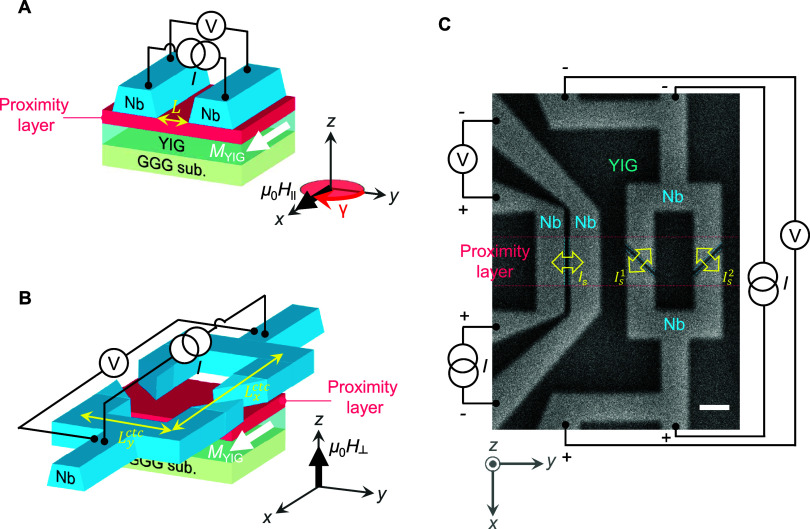
Josephson φ_0_-junction and SQUID fabricated on the same proximity layer. (A) Schematic illustration of the Josephson φ_0_-junction and measurement configuration. Here, *L* is the length of the Josephson φ_0_-barrier. Notice that γ is the azimuthal angle of the magnetization of the φ_0_-barrier (// μ_0_ *H*_∥_ = IP magnetic field) in the clockwise direction with respect to the *d.c*. bias current *I* along the *+y* axis. (B) Schematic of the superconducting quantum interferometer device (SQUID) and measurement scheme. Here, *L*_*x*/*y*_^ctc^ is the center-to-center spacing between the tracks defining the two opposite sides of the SQUID. (C) Scanning electron micrograph of the fabricated Josephson φ_0_-junction and SQUID on top of the same proximity layer, which is proximity-magnetized by a ferrimagnetic insulator Y_3_Fe_5_O_12_ (YIG) underneath it. Note that in the SQUID geometry, supercurrent flow directions (*I*_s_^1^, *I*_s_^2^) of the constituent Josephson φ_0_-junctions are orthogonal to each other, allowing for measurements of both nonvolatile φ_0_ and zero-field supercurrent diode efficiency *Q*_μ_0_ *H* = 0_ in a single SQUID [12,20]. In (C), the scale bar is 1 μm.

## Results and Discussion

We first investigate how the zero-field diode properties rely on the choice of a proximity layer for the Josephson φ_0_-barrier. Figure 2A exhibits *zero-field* current–voltage *I–V* curves of the magnetic 4-nm-thick Pt JJ, normalized by $I_{c}^{\mathrm{avg}}=\frac{I_{c}^{+}+\left|I_{c}^{-}\right|}{2}$ , for two different magnetization orientations *M*_Pt_ (// ± *x*-axis) below the junction’s superconducting transition temperature *T*_c_, taken at the temperature *T* = 2 K. As consistent with our recent experiment,^7^ visible zero-field supercurrent nonreciprocity Δ*I*_c, μ_0_ *H* = 0_ = *I*_c, μ_0_ *H* = 0_^+^ – |*I*_c, μ_0_ *H* = 0_^–^| ≠ 0 is detected and its polarity is clearly reversed (Δ*I*_c, μ_0_ *H* = 0_ > 0 → Δ*I*_c, μ_0_ *H* = 0_ < 0) when *M*_Pt_ flips from the positive to the negative *x*-direction, further proving the zero-field, polarity-switchable Josephson supercurrent nonreciprocity in the exchange-spin-split Pt JJs.^5^ By replacing the Pt Josephson φ_0_-barrier with a 5d heavy metal (HM = Ta or W) layer in Figure 2B,C, we find two intriguing HM-dependent diode properties. First, the zero-field diode efficiency $\left|Q_{\mu_{0}H=0}\right|=\left|\frac{{\Delta I}_{c,\mu_{0}H=0}}{2I_{c,\mu_{0}H=0}^{\mathrm{avg}}}\right|\approx 17\%$ obtained from the 4 nm-thick Ta JJ (Figure 2B) is comparable to that of the 4-nm-thick Pt JJ (Figure 2A), whereas the 4-nm-thick W JJ (Figure 2C) reveals a much smaller |*Q*_μ_0_ *H* = 0_| ≈ 5%. Second, the zero-field diode polarity *Q*_μ_0_ *H* = 0_ > 0 (*Q*_μ_0_ *H* = 0_ < 0) of the Ta and W JJs appears *opposite* to that *Q*_μ_0_ *H* = 0_ < 0 (*Q*_μ_0_ *H* = 0_ > 0) of the Pt JJs for the positive (negative) *x*-direction remanent-state *M*_HM_.

**Figure 2:**
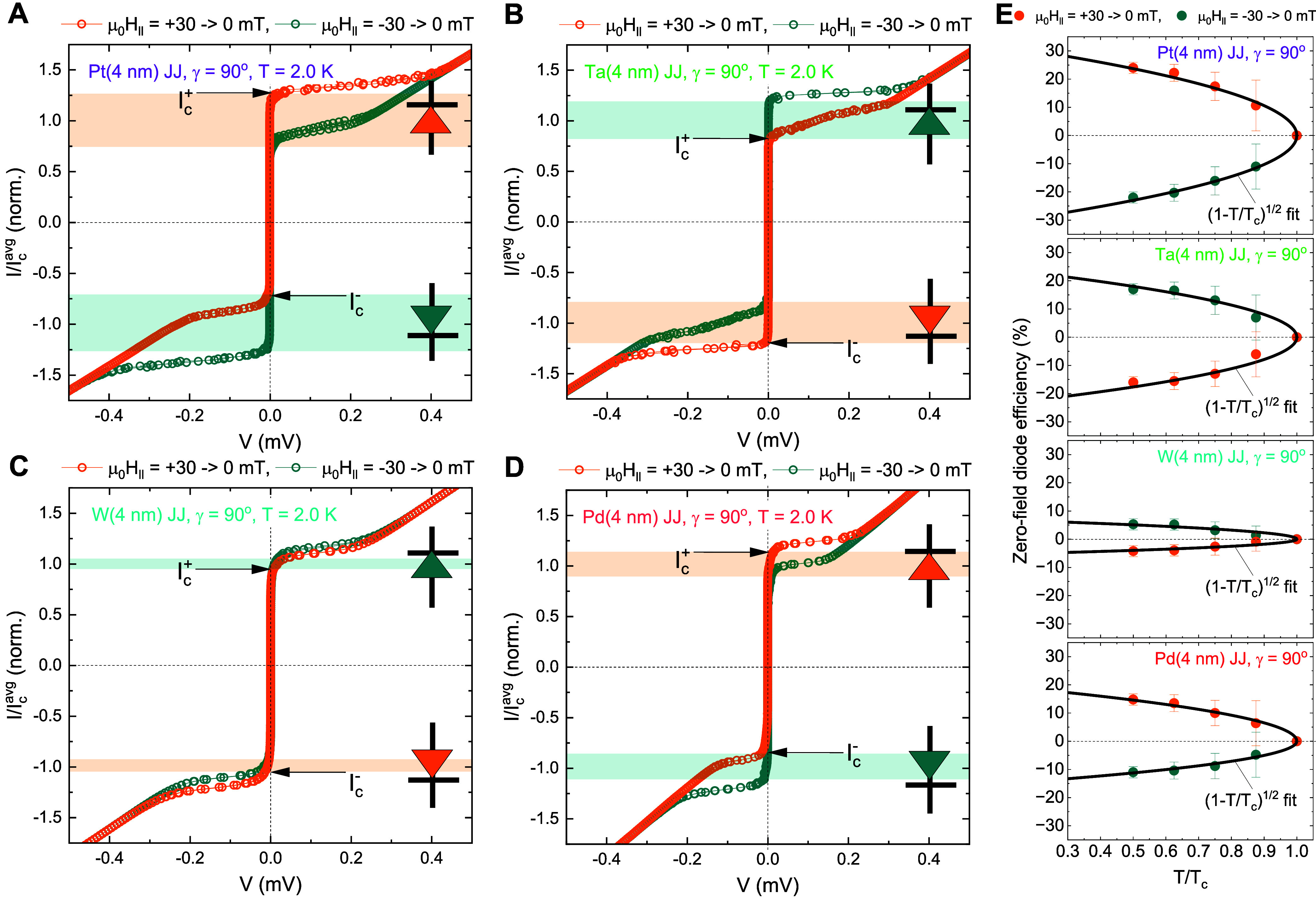
Zero-field polarity-switchable Josephson diodes with a different proximity layer. (A) Zero-field current–voltage *I–V* curves of the magnetic 4-nm-thick Pt Josephson junction (JJ) for two different Pt magnetization *M*_Pt_ (// ± *x*-axis) below the junction’s superconducting transition temperature *T*_c_. Here, *I* is normalized by $I_{c}^{\mathrm{avg}}\left(=\frac{I_{c}^{+}+\left|I_{c}^{-}\right|}{2}\right)$ = 70–105 μA. In the yellow (cyan) shaded regime, the Josephson supercurrent flows only in the positive (negative) *y*-direction, as indicated by the diode symbols. Notice that the displayed *I–V* curves were obtained by sweeping *I* forward and backward, *e.g.*, 0 mA → + 0.15 mA → −0.15 mA → 0 mA. (B, C) Data equivalent to (A) but for the JJ with a different 5d HM barrier. In (B, C), the 4-nm-thick Ta (W) proximity layer is employed. Note that the zero-field diode polarity of Ta and W JJs is found to be opposite to that of Pt JJ. (D) Data equivalent to (A) but for the JJ with a 4d Pd­(4 nm) proximity layer with high (para-)­magnetic susceptibility (30,31). (E) Zero-field diode efficiency $Q_{\mu_{0}H=0}={\frac{{\Delta I}_{c}}{{2I}_{c}^{\mathrm{avg}}}|}_{\mu_{0}H=0}$ as a function of normalized temperature *T*/*T*_c_ for the Pt, Ta, W, and Pd JJs.

To understand these, we have developed our own theoretical model, analyzing a metal with a Rashba spin–orbit interaction connected to two SCs and a ferromagnetic insulator. Using the Usadel equations, we describe the propagation of both singlet and triplet superconducting correlations in the diffusive limit (see the Supporting Information for full details). From this analysis, we find that

$$Q_{\mu_{0}H=0}\propto \varphi_{0}=-\mathrm{\alpha ̃}{\mathrm{\gamma ̃}}_{\phi }L^{2}=-\frac{\alpha \gamma_{\phi }}{\sqrt{8m^{2}D^{3}\hbar \left|\omega_{n}\right|}}L^{2}\approx -\frac{\alpha \gamma_{\phi }}{\sqrt{8m^{2}D^{3}\hbar \left|\pi k_{B}T\right|}}L^{2}$$

with γ = 90 ° for a diffusive (*l*_mfp_ < ξ, *l*_mfp_ < *L*) junction. Here, $\mathrm{\alpha ̃}=\frac{\alpha }{2\mathrm{mD}}$ and ${\mathrm{\gamma ̃}}_{\phi }=\frac{\gamma_{\phi }}{\sqrt{2\hbar D\left|\omega_{n}\right|}}$ . α is the Rashba coefficient, *m* is the effective electron mass, *D* is the diffusion coefficient, γ_ϕ_ = −Δ*E*_ex_ represents the exchange-spin-splitting, *ℏ* is the reduced Planck constant, ω_n_ (≈ π*k*_B_ *T* in the high *T* limit) is the Matsubara frequency, and γ is the azimuthal angle (Figure 1A). *l*_mfp_, ξ, and *L* are the mean free path, coherence length, and spacing of the Josephson φ_0_-barrier (Figure 1A), respectively. Since the electrical resistivity of Pt, Ta, W, and Pd layers, which is related with *m* and *D*, does not significantly differ among these materials (Figure S3) and *L* is fixed at 80–100 nm (see the Methods and Experimental Section), it is reasonable to speculate that γ_ϕ_ and α predominantly govern the polarity and amplitude of the Josephson diode efficiency over *m* and *D*. Earlier theories,^28,29^ in fact, pointed out that the *magnitude and sign* of γ_ϕ_ are strongly dependent on the interface properties.

Based on the results of the anomalous Hall response (Figure S3) and nonlocal thermal magnon transport (Figure S4), which respectively characterize the magnetic proximity effect and the spin-to-charge conversion, we now explain the two aforementioned properties: the proximity-layer-dependent diode strength and polarity. The W layer exhibits a smaller anomalous Hall response in Figure S3 (while showing a comparable spin-to-charge conversion efficiency in Figure S4) compared to the Pt and Ta layers. Given this, the first property is explained in terms of the weak proximity-induced Δ*E*_ex_ in the W layer, as in line with its very low (para-)­magnetic susceptibility.^28^ This explanation can also be supported by a large |*Q*_μ_0_ *H* = 0_| ≈ 15% (Figure 2D) attained from the JJ with a highly magnetic-susceptible Pd layer^30,31^ (Figure S3) having a modest SOC strength (Figure S4). The second property relates to how the overall sign of γ_ϕ_α is given in our structure, in which the HM Josephson barrier is sandwiched between top AlO_*x*_ and bottom YIG layers (see the Methods for details). The sign of γ_ϕ_ (= −Δ*E*_ex_) relies on whether the magnetic proximity coupling^32^ of the HM is interlayer-ferromagnetic or interlayer-antiferromagnetic with the YIG magnetization, whereas the sign of α is determined by the sum of potential gradient^27,32^ at the top AlO_*x*_/HM interface and that at the bottom HM/YIG interface. This indicates that the proximity magnetic ordering across the bottom HM/YIG interface and the Rashba effect at the *top* AlO_*x*_/HM interface are both responsible for the sign-reversed diode effect in the Ta and W JJs. In the framework of standard spin pumping theory, interfacial spin-transfer is typically described by a complex quantity known as the spin mixing conductance *G*_↑↓_= *G*_*r*_ + *iG*_*i*_, where the transfer of the interfacial exchange field is included in the imaginary part of the spin mixing conductance, *G*_*i*_. We also note that the sign of γ_ϕ_ (= −Δ*E*_ex_), which reflects whether the HM/YIG coupling is interlayer-ferromagnetic or -antiferromagnetic, is theoretically linked to the sign of *G*_*i*_. However, to the best of our knowledge, there is no direct experimental demonstration showing that a sign change in *G*_*i*_ necessarily guarantees an inversion of γ_ϕ_ (= −Δ*E*_ex_), leaving this to be firmly confirmed in future studies.

By plotting *Q*_μ_0_ *H*=0_ as a function of *T*/*T*_c_ for four different JJs (Figure 2E), we witness the progressive enhancement of *Q*_μ_0_ *H* = 0_ with decreasing *T*/*T*_c_, and for all the JJs, *Q*_μ_0_ *H* = 0_ (*T*/*T*_c_) is well fitted by a $\sqrt{1-\frac{T}{T_{c}}}$ function. This proximity-layer-independent *T*-evolving behavior provides an experimental signature of the exchange-spin-split Josephson φ_0_-junctions^24–26^ . In fact, our theory predicts that as *T*/*T*_c_ goes down, Δ*E*_ex_ and α in the numerator [*D* and ω_n_ (≈ π*k*_B_ *T*) in the denominator] of φ_0_ increase (decrease), which qualitatively explains the *effective* enhancement of $Q_{\mu_{0}H=0}\propto \varphi_{0}\;\propto \;\frac{\gamma_{\phi }\alpha }{D^{1.5}\sqrt{T}}$ (for γ = 90 °) at a lower *T*/*T*_c_. Furthermore, the predicted and experimentally observed reduction of higher harmonics with increasing *T*/*T*_c_ ^33^, which suppresses the nonreciprocity $Q\approx -\frac{I_{c2}\varphi_{0}}{I_{c1}}$ at higher *T*/*T*_c_, is well consistent with our observations.

If the Josephson φ_0_-barrier with Rashba SOC is nonmagnetic,^14–22^ *Q* scales linearly with the strength of in-plane (IP) magnetic field μ_0_ *H*_∥_ in the low-field regime, i.e., magneto-linearity.^14,16,20^ On the other hand, if it is proximity-magnetized,^24–26^ *Q* (μ_0_ *H*_∥_) mimics the μ_0_ *H*_∥_-driven reversal of the φ_0_-barrier’s magnetization *M*_φ_0_–barrier_, i.e., magneto-hysteresis.^5,7^ This hysteretic characteristic of exchange-spin-split φ_0_-junctions can be seen in the *Q* (μ_0_ *H*_∥_) plots of our Pt, Ta, W, and Pd JJs at a fixed γ = 90 ° (Figure 3A–3D). Here, all the junctions exhibit the low-field magneto-hysteresis for |μ_0_ *H*_∥_| ≤ 5 mT, above which |*Q*| diminishes monotonically, regardless of the diode polarity. We note that for Zeeman-spin-split φ_0_-junctions,^17–20^ the magnetic-field-dependent Δ*I*_c_(μ_0_ *H*_∥_) is controlled by the two competing effects^17^ of (1) the anomalous phase shift φ_0_(μ_0_ *H*_∥_) ∝ μ_0_ *H*_∥_ versus (2) the ratio of first and second harmonic terms $\frac{I_{c2}\left(\mu_{0}H_{∥}\right)}{I_{c1}\left(\mu_{0}H_{∥}\right)}\propto \frac{\Delta \left(\mu_{0}H_{∥}\right)}{\Delta_{0}}$ , where Δ­(μ_0_ *H*_∥_) is the suppressed superconducting energy gap in the presence of μ_0_ *H*_∥_ and Δ_0_ is the zero-field superconducting gap *at the Josephson barrier*. Since in our exchange-spin-split φ_0_-junctions, the internal exchange field dominates the necessary spin-splitting of the Josephson barrier (or, proximity layer) over the external magnetic field, one can assume that (1) φ_0_(μ_0_ *H*_∥_) remains constant and consequently, conclude that (2) the pair breaking factor $\frac{I_{c2}\left(\mu_{0}H_{∥}\right)}{I_{c1}\left(\mu_{0}H_{∥}\right)}\propto \frac{\Delta \left(\mu_{0}H_{∥}\right)}{\Delta_{0}}$ *at the Josephson barrier* is responsible for the monotonic decrease of Δ*I*_c_(μ_0_ *H*_∥_) observed in our φ_0_-junctions (Figure 3). A rather slow suppression of |*Q*| for the W JJ in |μ_0_ *H*_∥_| > 5 mT (Figure 3C) is also understood in terms of a non-negligible relative contribution of Zeeman-spin-splitting to the *weak* exchange-spin-splitting induced in the W proximity layer (Figure S3).

**Figure 3:**
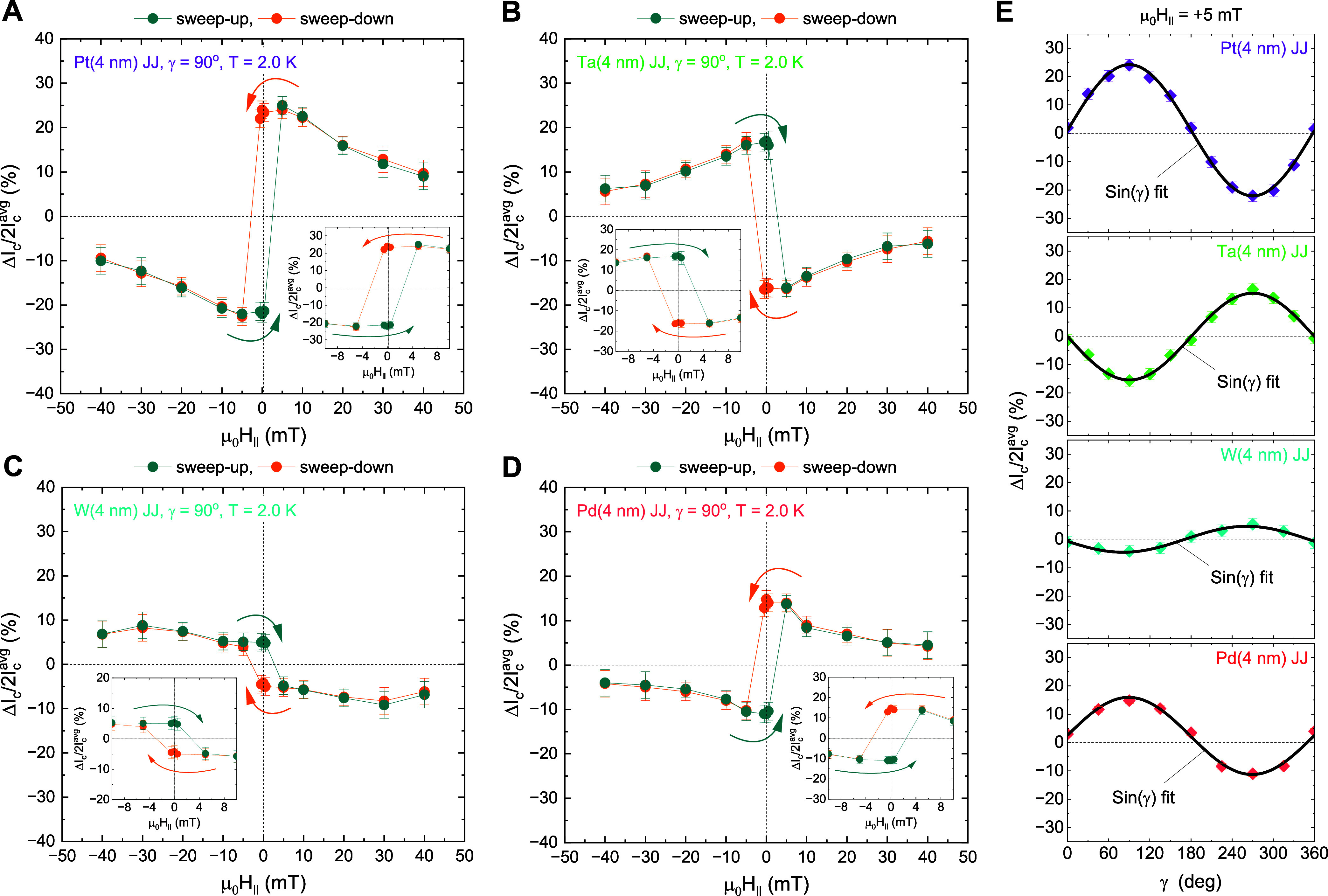
Magnetic field strength and angle dependences of the Josephson diode efficiency. (A) Josephson diode efficiency $Q=\frac{{\Delta I}_{c}}{{2I}_{c}^{\mathrm{avg}}}\left(=\frac{I_{c}^{+}-\left|I_{c}^{-}\right|}{I_{c}^{+}+\left|I_{c}^{-}\right|}\right)$ versus in-plane (IP) magnetic-field-strength μ_0_ *H*_∥_ plot for the magnetic 4-nm-thick Pt Josephson junction (JJ), taken at the fixed azimuthal angle γ = 90 ° of the Pt magnetization *M*_Pt_ with respect to the positive *y*-axis. Notice that the orange and cyan symbols represent, respectively, the sweep-up and sweep-down directions of μ_0_ *H*_∥_, and the error bars for the applied μ_0_ *H*_∥_ (approximately 0.01 mT) are much smaller than the size of the data points. The inset shows a magnified plot around μ_0_ *H*_∥_ = 0. (B, C) Data equivalent to (A) but for the JJ with a different 5d HM layer. In (B, C), the 4-nm-thick Ta (W) proximity layer is used. (D) Data equivalent to (A) but for the JJ with a 4d Pd­(4 nm) proximity layer with high (para-)­magnetic susceptibility (30,31). (E) γ-dependent $\frac{{\Delta I}_{c}}{{2I}_{c}^{\mathrm{avg}}}$ of the Pt, Ta, W, and Pd JJs at the constant μ_0_ *H*_∥_ = 5 mT, which is applied to rotate *M*_Pt_ (// μ_0_ *H*_∥_) in the plane. Since the strength of exchange-spin-splitting fields in our proximity system is greater than μ_0_ *H*_∥_ = 5 mT, φ_0_ can be assumed to be constant for this measurement.

The magneto-chirality, *Q* ∝ φ_0_ × sin­(γ) in the limit of a small second-harmonic contribution and small φ_0_,^5,7,14,16,20^ another characteristic of the Josephson φ_0_-junctions^14–22,24–26^ with isotropic SO fields (e.g., Rashba SOC), is also checked by measuring *Q* as a function of γ for our Pt, Ta, W, and Pd JJs at a constant μ_0_ *H*_∥_ = 5 mT. As summarized in Figure 3E, for all the JJs, the *Q*_μ_0_ *H*_∥_ = 5 mT_(γ) data are well fitted by a sine function, further supporting an intrinsic φ_0_-origin^14–22,24–26^ of their Josephson supercurrent nonreciprocity.

To directly probe the nonvolatile φ_0_ in our exchange-spin-split JJs and to correlate it with *Q*_μ_0_ *H* = 0_, we next execute the phase-resolved interferometry characterization using a direct-current (d.c.) SQUID^12,17–20^ in the voltage state.^20,34^ Our SQUID geometry is designed for supercurrents (*I*_s_^1^, *I*_s_^2^) of two symmetric Josephson φ_0_-junctions to flow orthogonal to each other (Figure 1C), enabling measurements of both nonvolatile φ_0_ and *Q*_μ_0_ *H* = 0_ in a single SQUID (12,20) (Figure S2). Note also that in our SQUID geometry, the nonzero *Q*_μ_0_ *H* = 0_ can be detected by setting the directions of *I*_s_^1^ and *I*_s_^2^, respectively, perpendicular and parallel to the remanent-state *M*_φ_0_–barrier_, or vice versa (Figure S5). When the SQUID, which is composed of overdamped JJs with no hysteresis (Figure 2A–2D) and in the limit of small self-inductance with negligible screening (see the Supporting Information for a quantitative discussion), is *I*-biased, the conversion of a magnetic flux into (time-averaged) *V* modulation for the ± *x*-direction remanent-state *M*_φ_0_–barrier_ in the low-field limit is given by^20,31^ $V_{\pm }\left(\mu_{0}H_{⊥},I\right)=\frac{R_{n}}{2}\sqrt{{\left(I\right)}^{2}-{\left(2I_{c}^{\mathrm{avg}}\;\cos\left(\pi \frac{\Phi_{\mathrm{SQUID}}}{\Phi_{0}}\pm \frac{\varphi_{\mathrm{tot}}}{2}\right)\right)}^{2}}$ . Here, we assume that two symmetric JJs constitute the SQUID, *R*_n_ (*I*_c_^avg^) is the normal-state zero-bias resistance (averaged critical current) of each JJ, Φ_SQUID_ = μ_0_ *H*_⊥_ *A*_SQUID_^eff^ is the magnetic flux threading the SQUID loop given by μ_0_ *H*_⊥_ and *A*_SQUID_^eff^ = *L*_*x*_^ctc^ *L*_*y*_^ctc^ ≈ 11 μm^2^ (Figure 1B,1C, ref 30), and $\Phi_{0}=\frac{h}{2e}=2.07\times 10^{-15}\;T\cdot m^{2}$ is the magnetic flux quantum. $\varphi_{\mathrm{tot}}=\varphi_{0}^{1}\;\sin\left(\frac{\pi }{4}\right)+\varphi_{0}^{2}\;\sin\left(\frac{\pi }{4}\right)\approx 2\varphi_{0}\;\sin\left(\frac{\pi }{4}\right)$ , where φ_0_^1^ (φ_0_^2^) is the anomalous phase shift for the first (second) constituent JJ, the prefactor 2 accounts for the doubled anomalous phases acquired by two symmetric JJs around the SQUID loop and the postfactor $\sin\left(\frac{\pi }{4}\right)$ takes the magneto-chiral effect into account (Figure S2). As previously pointed out,^17–20^ it is challenging to accurately determine the absolute φ_0_ of the constituent JJs due to a nonvanishing screening effect^35^ in the SQUID loop under application of μ_0_ *H*_⊥_. Therefore, we below focus our analysis on the horizontal difference of *V*_+_ (μ_0_ *H*_⊥_, *I*) versus *V*_–_ (μ_0_ *H*_⊥_, *I*), which provides a reliable measure of $\varphi_{0}=\frac{\varphi_{\mathrm{tot}}}{2\;\sin\left(\frac{\pi }{4}\right)}$ .

Clearly, as presented in Figure 4A–H, *V*_–_ (μ_0_ *H*_⊥_) horizontally shifts with respect to *V*_+_ (μ_0_ *H*_⊥_), and the details depend on the type of constituent JJs. Qualitatively, the relative horizontal shift is positive (negative) for the Pt JJ-based (Ta JJ-based) SQUID, while it is almost unchanged for the W JJ-based SQUID. Moreover, the *V*_±_ (Φ_SQUID_/Φ_0_) data of Pd JJ-based SQUID indicate the positive relative horizontal shift, which is the same as that of the Pt JJ-based SQUID. Best fits to the measured *V*_±_ (Φ_SQUID_/Φ_0_) data (Figure 4A–C) using the above formula give φ_tot_ = (+0.62 ± 0.03) π, (−0.46 ± 0.03) π, (−0.04 ± 0.02) π and (+0.41 ± 0.01) π, corresponding to φ_0_ = (+0.44 ± 0.02) π, (−0.33 ± 0.02) π, (−0.03 ± 0.01) π and (+0.29 ± 0.01) π, respectively, for the Pt JJ-based, Ta JJ-based, W JJ-based and Pd JJ-based SQUIDs. By comparing and correlating these measured (nonvolatile) φ_0_ with the zero-field Josephson supercurrent nonreciprocity (Figures 2 and S5), we provide strong evidence for a direct link between these in our exchange-spin-split JJs. In particular, the estimated large values of φ_0_ = +0.44 ± 0.02π (+0.29 ± 0.01π) and −0.33 ± 0.02π, respectively, for the Pt (Pd) and Ta JJs can account for their notably large *Q*_μ_0_ *H* = 0_ with the opposite diode polarity. As shown in Figure 5, the empirical linear scaling between the measured φ_0_ and the obtained *Q*_μ_0_ *H* = 0_ provides strong evidence that φ_0_ is indeed the dominant factor contributing to *Q*_μ_0_ *H* = 0_. According to recent theories,^36,37^ the low-*T* limit (*T* < 0.1 *T*_c_) supercurrent diode efficiency of *intrinsic origin* can reach 20–30% at optimal magnetic fields, spin–orbit coupling, and junction geometry. In this regard, we find that the *zero-field* diode efficiency of ≥15% obtained in our exchange-spin-split φ_0_-junctions at 2 K (*T* ∼ 0.5 *T*_c_) is promising.

**Figure 4:**
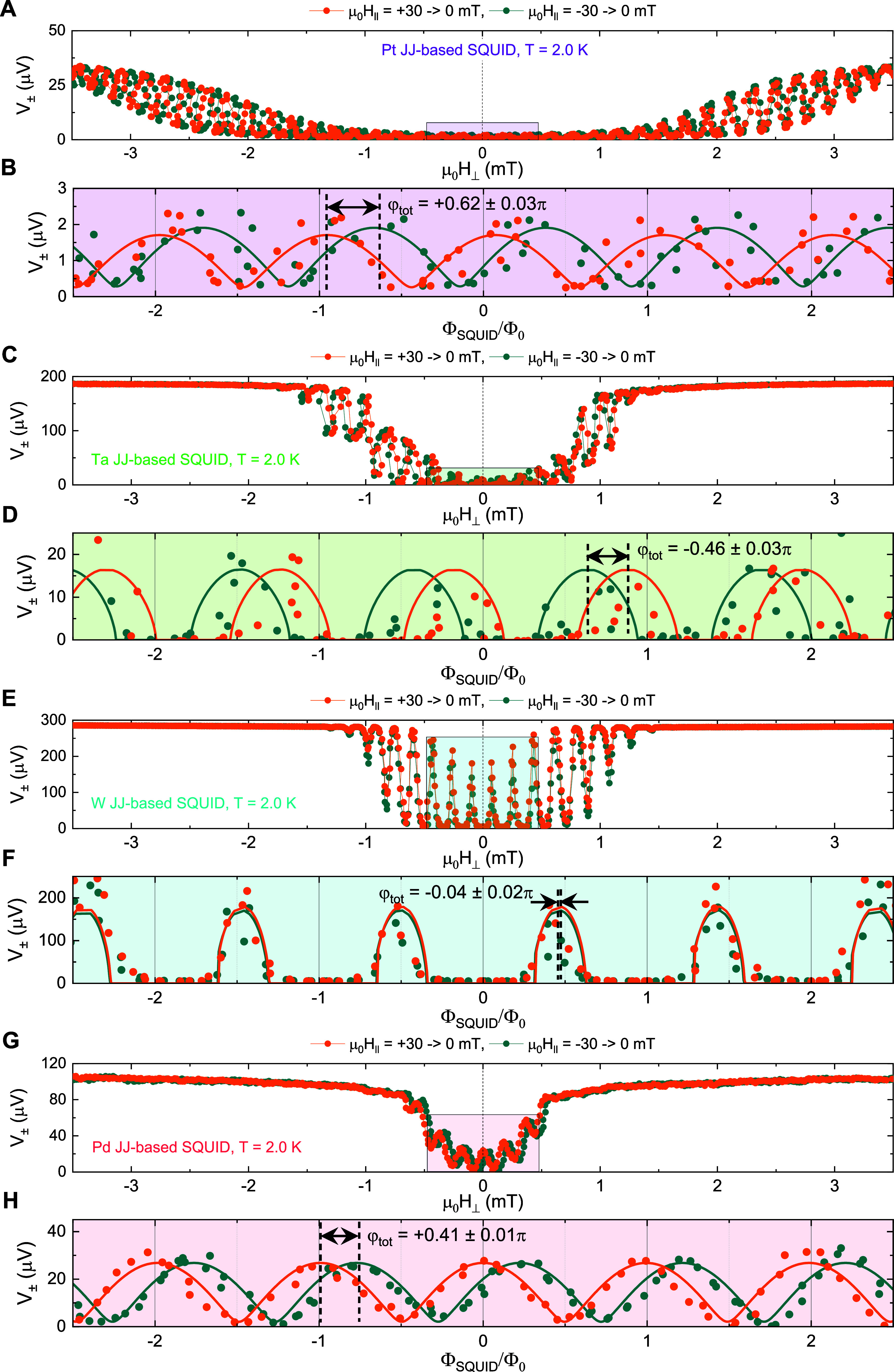
Probing φ_0_ by SQUIDs and its correlation with the zero-field diode efficiency. (A) Time-averaged voltage *V*_±_ as a function of perpendicular magnetic field μ_0_ *H*_⊥_ for the d.c. current *I*-biased SQUID, which consists of Pt­(4 nm) JJs. Here, the applied d.c. bias *I* is in the range of 145–210 μA. (B) Low-field *V* oscillation as a function of normalized magnetic flux Φ_SQUID_/Φ_0_, corresponding to the wine shaded regime in (A). (C, D) Data equivalent, respectively, to (A, B) but for the SQUID with Ta­(4 nm) JJs. (E, F) Data equivalent, respectively, to (A, B) but for the SQUID with W­(4 nm) JJs. (G, H) Data equivalent, respectively, to (A, B) but for the SQUID with Pd­(4 nm) JJs. Notice that for the Pd JJ-based SQUID, a non-negligible parabolic-like background signal is subtracted from (G) to better estimate the nonvolatile φ_0_ of our magnetic Pd JJs in (H).

**Figure 5:**
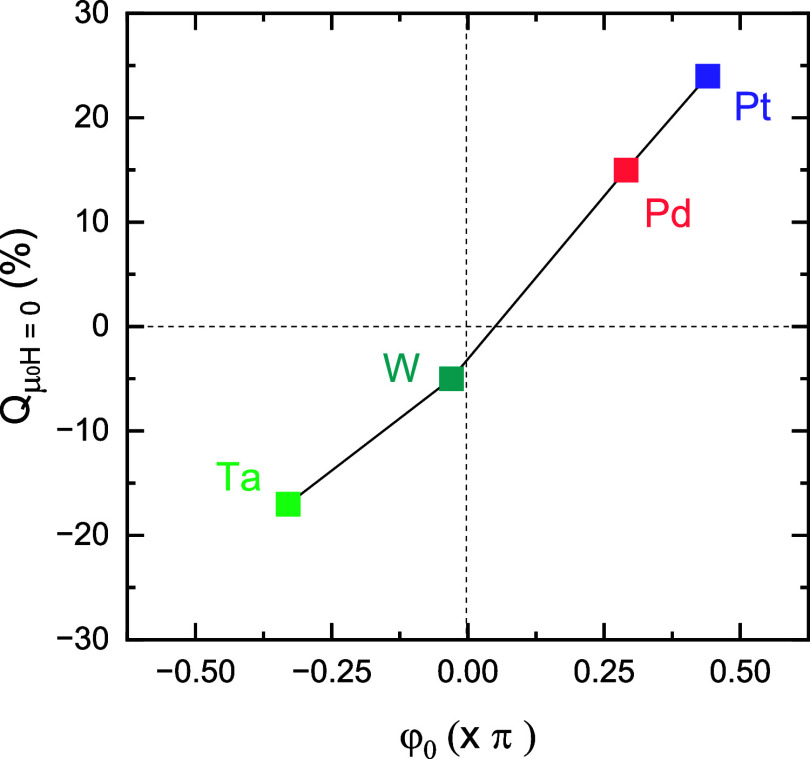
Proportionality of φ_0_ to the zero-field diode efficiency *Q*_μ_0_ *H* = 0_. The obtained *Q*_μ_0_ *H* = 0_ (Figure 2) is plotted as a function of the measured φ_0_ (Figure 4), revealing a clear empirical linear relationship. Note that the error bars for φ_0_ and *Q*_μ_0_ *H* = 0_ are smaller than the size of the data points.

We finally compare our results with a previous observation of the nonvolatile φ_0_ in SQUIDs made with symmetric Al/InAs nanowire/Al Josephson junctions (JJs).^12^ In that case, unpaired spins arising from surface oxides or defects act as ferromagnetic impurities, giving rise to φ_0_ nonvolatility. The earlier nanowire φ_0_ experiments thus rely on localized impurity spins interacting with the superconducting phase, which is fundamentally different from our approach that leverages engineered proximity magnetization combined with Rashba SOC. Although the observed φ_0_values are of the order of π, similar to ours, the weak controllability of surface spin density, spatial distribution, and spin orientation in the nanowire structure limits its practical prospects for large-scale integration. In contrast, while achieving reproducible interfacial magnetic ordering and controlled Rashba fields across wafers remains challenging and requires careful materials optimization, our work demonstrates that using conventional spintronic and superconducting materials with industry-compatible sputter deposition provides a higher degree of tunability and compatibility with wafer-scale fabrication.

## Conclusions

On the basis of exchange-spin-split JJs^24–26^ and superconducting quantum interferometry,^12,17–20^ we have experimentally identified the proximity role of the Josephson φ_0_-barrier in determining the zero-field supercurrent diode properties and demonstrated the presence of φ_0_ ^20–22^ without μ_0_ *H* and its direct link with the zero-field diode effect. We believe that our interferometry characterization of the nonvolatile φ_0_ provides a timely and important step for confirming the intrinsic φ_0_-origin of the nonreciprocal Josephson supercurrents, for the φ_0_-tuning of the zero-field diode performance (i.e., sign and magnitude), and for devising a new generation of the cryogenic nonvolatile φ_0_-memory. We expect that our results will provide a guideline for further technical improvements through the right choice of the proximity layer for the Josephson φ_0_-barrier, as also pointed out in recent theories.^36,37^ Given the recent adoption of vortex-driven supercurrent rectifiers^38,39^ in AC-to-DC converters with active lateral dimensions on the order of several micrometers for logic circuit applications, our φ_0_-tunable Josephson diodes can enable the realization of ultralow-power, high-density circuitry, as the operating principle of our devices is free from the geometric constraints^38,39^ required to accommodate Josephson or Abrikosov vortices and to induce their rectifying motion.

## Methods and Experimental Section

### Sample Growth and Device Fabrication

Four different types of proximity layers of Pt­(4–15 nm), Ta­(4–15 nm), W­(4–15 nm), and Pd­(4–15 nm) were first sputter-grown on single-crystalline YIG­(∼200 nm) films (5) (Figure S1) at room temperature by d.c. magnetron plasma sputtering in an ultrahigh vacuum system with a base pressure of 1 × 10^–9^ Torr. All these films were sputtered at 27 °C with a sputter power of more or less 15 W and at an Ar pressure of 3 mTorr and were capped with a naturally oxidized AlO_*x*_(1 nm) layer that was formed by sputter deposition of Al to prevent oxidation of the proximity layer.

To fabricate the lateral JJ and SQUID (Figure 1A–C), a central track of the proximity layer with lateral dimensions of 1.5 × 50 μm^2^ is first defined using optical lithography, Ar^+^-ion beam etching. We then defined electrical leads and bonding pads, formed from Au­(60–80 nm)/Ru­(2 nm), which were deposited by Ar^+^-ion beam sputtering. Multiple Nb electrodes to form the lateral JJ and SQUID were subsequently defined on top of the central track via electron-beam lithography and lift-off steps. The Nb­(50–60 nm) electrodes were grown by Ar^+^-ion beam sputtering at an Ar pressure of 1.5 × 10^–4^ mbar. Before sputtering the Nb electrodes, the AlO_*x*_ capping layer and Au surface were Ar-ion beam-etched away to make direct metallic electrical contacts. All lift-off processes were done with acetone in ultrasonic baths. We note that the edge-to-edge spacing *L* between the adjacent Nb electrodes of the lateral JJ (Figures 2 and 3) and SQUID (Figures 4 and S5) was fixed at 80–100 nm to make the Josephson nonreciprocal supercurrents and anomalous phase shift (*Q* ∝ φ_0_ ∝ *L* ^2^, see Supporting Information for details) detectably large at 2 K in a ^4^He cryostat.

### Measurements and Analysis of Josephson *I–V* Curves and SQUID Data

We measured current–voltage *I*–*V* curves of the fabricated JJs and SQUIDs (Figures 2, 3, and S5) and voltage-flux *V*–Φ_SQUID_/Φ_0_ curves of the *I*-biased SQUIDs (Figures 4) with a four-probe configuration in a Quantum Design Physical Property Measurement System (PPMS) using a Keithley 6221 current source and a Keithley 2182A nanovoltmeter. We carried out the demagnetization process of an unintentionally trapped magnetic flux in superconducting coils of the PPMS for >3 h at *T* > 10 K in advance of the measurements.

For the *zero-field I*–*V* curve measurements (Figures 2 and S5) below the junctions’ *T*_c_ (< 6 K), μ_0_ *H*_∥_ = ±30 mT, much larger than the coercive field of YIG^5^, is first applied along the *x*-axis and then returned to zero to preconfigure the remanent-state *M*_φ_0_–barrier_. The Josephson critical current *I*_c_ was determined by fitting the measured *I*–*V* curves with the standard formula for overdamped junctions,^34^ $V\left(I\right)={\frac{I}{\left|I\right|}R}_{n}\sqrt{I^{2}-I_{c}^{2}}$ . When thermal noise/rounding effects^34^ $\left(\propto \frac{T}{I_{c}}\right)$ on the *I*–*V* curves become significant, especially for μ_0_ *H*_∥_ ≥ 30 mT or *T* ≥ 3.5 K, we determined the *I*_c_ value at the point where *V* (*I*) ≈ 1 μV. We obtained the field-strength and field-angle dependences of *I*_c_ (μ_0_ *H*_∥_) and *I*_c_ (γ) (Figure 3) by repeating the *I*–*V* curve measurements at *T* = 2 K at the applied μ_0_ *H*_∥_, parallel to the interface plane of Nb electrodes. For a proximity layer with a fixed thickness of 4 nm, we conducted *I–V* measurements on at least five different JJs on the same YIG/GGG(111) substrate. We found that over 80% of the working devices exhibited the same sign and a similar magnitude (±10%) of zero-field Josephson diode efficiency.

We measured the *V*_±_ (μ_0_ *H*_⊥_,*I*) curves for the *I*–biased SQUID by sweeping μ_0_ *H*_⊥_ up and down up to |μ_0_ *H*_⊥_| = 4 mT. Note that as shown in our previous experiment^35^ with a Cu JJ-based SQUID, the field drift error was found to be quite small (∼0.01 mT). In Figure 4, the measured *V*_±_ (Φ_SQUID_/Φ_0_) data appear somewhat noisy. This noise is likely due to the challenge of precisely controlling the magnetic field at ∼0.01 mT using a superconducting electromagnet in the PPMS, rather than issues with the amplitude of the voltage signals. Note also that given that the typical coercive field of YIG­(∼200 nm) fims at 2 K is on the order of 0.1 mT, and considering that the patterned proximity-magnetized Pt layer generally exhibits a larger coercive field than YIG, we can rule out the presence of unintentionally trapped flux (on the order of 0.01 mT) at μ_0_ *H*_∥_ = 0 mT as a potential cause for the zero-field supercurrent diode effect and its polarity reversal in our system.

## Supplementary Material



## Data Availability

All data needed to evaluate the conclusions in the paper are present in the paper and/or the Supporting Information.

## Notes

Analytical expression for the diode efficiency in the limit of a small second harmonic and small φ_0_; a quasiclassical theory of diffusive superconducting φ_0_ Josephson junctions with a proximized Rashba normal metal and a spin-active interface with a magnetic insulator; quantitative estimates of loop inductance and flux offset; x-ray structural analysis of Josephson barriers; comparison of measurement configurations; estimation of exchange spin-splitting from anomalous Hall effect measurements; characterization of spin-orbit coupling from non-local thermal-magnon transport measurements; zero-field polarity-switchable supercurrent diode effect detected in the SQUID consisting of symmetric JJs; and comparison between exchange–spin-split (this work) and Zeeman–spin-split (field-driven) φ_0_ junctions (PDF)
