# Supplementary material for: Interferometric Evidence of Nonvolatile Anomalous Phase Shifts in Exchange-Spin-Split Josephson Supercurrent Diodes
Source: ACS Nano. 2026 Jan 22;20(5):4384–92. doi: 10.1021/acsnano.5c17979 (PMC12895565; doi:10.1021/acsnano.5c17979)
Supplement: Supplementary file 1 [file nn5c17979_si_001.pdf]

Supplementary Material

Interferometric evidence of non-volatile anomalous phase shifts in exchange-spin-split Josephson supercurrent diodes

Kun-Rok Jeon,[1*†] Jae-Keun Kim,[2†] Jiho Yoon,[2] Jae-Chun Jeon,[2] Hyeon Han,[2,3]

Audrey Cottet,[4,5] Takis Kontos,[4,5] and Stuart S. P. Parkin[2*]

[1]*Department of Physics, Chung-Ang University (CAU), 06974 Seoul, Republic of Korea*

[2]*Max Planck Institute of Microstructure Physics, Weinberg 2, 06120 Halle (Saale), Germany*

[3]*Department of Materials Science and Engineering, Pohang University of Science and Technology (POSTECH), 37673 Pohang, Republic of Korea*

[4]*Laboratoire de Physique de l'Ecole Normale Supérieure, ENS, Université PSL, CNRS, Sorbonne Université, Université Paris-Diderot, Sorbonne Paris Cité, 75005 Paris, France.*

[5]*Laboratoire de Physique et d'Etude des Matériaux, ESPCI Paris, PSL University, CNRS, Sorbonne Université, 75005 Paris, France.*

*Corresponding author. Email: jeonkunrok@gmail.com (K.-R.J.); stuart.parkin@halle-mpi.mpg.de (S.S.P.P.)

[†]These authors contributed equally to this work.

This PDF file includes:

Supplementary Text

Figs. S1 to S6

Table S1

References (*S1-S19*)

Analytical expression for the diode efficiency in the limit of a small second-harmonic and small φ_0.

Josephson diode efficiency is defined as $Q = \frac{I_c^+ - |I_c^-|}{I_c^+ + |I_c^-|}$, where $I_c^+ = \max_{\varphi} I(\varphi)$ and $I_c^- = \min_{\varphi} I(\varphi)$. For the current-phase relation (CPR), $I(\varphi) = I_{c1}\sin(\varphi) + I_{c2}\sin(2\varphi + \varphi_0)$, taking the derivative with respect to φ gives $\frac{\mathrm{d}I(\varphi)}{\mathrm{d}\varphi} = I_{c1}\cos(\varphi) + 2I_{c2}\cos(2\varphi + \varphi_0) = 0$, which allows one to calculate the extrema I_c^+ and I_c^- numerically.

For small $I_{c2} \ll I_{c1}$, as is the case for our diffusive Josephson junctions, we can approximate the maxima and minima of $a_1\sin(\varphi)$ as occurring at $\varphi \approx \pi/2$ and $\varphi \approx -\pi/2$, respectively:

Maximum ($\varphi_{max} \approx \pi/2$): $I_c^+ \approx I_{c1}\sin\left(\frac{\pi}{2}\right) + I_{c2}\sin(\pi + \varphi_0) = I_{c1} - I_{c2}\sin(\varphi_0)$,

Minimum ($\varphi_{min} \approx -\pi/2$): $I_c^- \approx I_{c1}\sin\left(-\frac{\pi}{2}\right) + I_{c2}\sin(-\pi + \varphi_0) = -I_{c1} - I_{c2}\sin(\varphi_0)$.

Hence, the diode efficiency becomes $Q = \frac{I_c^+ - |I_c^-|}{I_c^+ + |I_c^-|} = \frac{I_{c1} - I_{c2}\sin(\varphi_0) - |-I_{c1} - I_{c2}\sin(\varphi_0)|}{I_{c1} - I_{c2}\sin(\varphi_0) + |-I_{c1} - I_{c2}\sin(\varphi_0)|} = -\frac{I_{c2}\sin(\varphi_0)}{I_{c1}}$. This provides a simple analytical expression for the diode efficiency in the small second-harmonic limit. Note that for small φ_0, where $\sin(\varphi_0) \approx \varphi_0$, the efficiency becomes $Q \approx -\frac{I_{c2}\varphi_0}{I_{c1}}$.

Quasiclassical theory of diffusive superconducting φ_0 - Josephson junction with a proximized Rashba normal metal and a spin active interface with a magnetic insulator.

We consider theoretically the experimental setup described in the main text. A metal with Rashba spin-orbit interaction is coupled to two superconducting electrodes and a ferromagnetic insulator. We derive the equations describing the propagation of superconducting correlations in the diffusive limit – Usadel equations. We consider here both singlet and triplet

superconducting correlations. We assume that the magnetization is along an axis transverse to the supercurrent flow, as sketched in Fig. 1A. This sets the magnetochiral term $\sin\gamma$ to 1. We keep this regime for the sake of simplicity in the following and focus on the combination of interface exchange field and Rashba spin orbit interaction for diffusive φ_0-Josephson junction physics, which is key to the non-volatile phase shift discussed in the main text.

We first consider a normal metal with Rashba spin-orbit interaction in contact with two superconducting electrodes. We take a Rashba spin-orbit term given by $\breve{H}_R = \frac{\alpha}{m}(\vec{\sigma} \wedge \vec{z}).\vec{p}$, where α being the Rashba coefficient and m is the effective electron mass. The minimal Hamiltonian then reads:

$$\widehat{H}_{N-Rashba} = \int d^3r \sum_{\sigma} \left(\psi_{\sigma}^{\dagger}(\vec{r}) \left[-\frac{\hbar^2\nabla^2}{2m} - \mu \right] \psi_{\sigma}(\vec{r}) \right)$$
$$-i\hbar\frac{\alpha}{m}\int d^3r \begin{bmatrix}\psi_{\uparrow}^{\dagger}(r) & \psi_{\downarrow}^{\dagger}(r)\end{bmatrix} \left(\breve{\sigma}_2\frac{\partial}{\partial x} - \breve{\sigma}_1\frac{\partial}{\partial y}\right) \begin{bmatrix}\psi_{\uparrow}(\vec{r}) \\ \psi_{\downarrow}(\vec{r})\end{bmatrix}$$

(S1)

where $\psi_{\sigma}^{\dagger}(r)$, $\psi_{\sigma}(\vec{r})$ are the field operators for electrons with spin $\sigma \in \{\uparrow,\downarrow\}$ at position $\vec{r}$. The operator $\vec{\sigma} = (\breve{\sigma}_1, \breve{\sigma}_2, \breve{\sigma}_3)$ is a spatial vector which encloses the spin Pauli matrices and μ is the chemical potential of the normal metal.

We now study the physical consequences of the above Hamiltonian. More precisely, our aim is to study the spatial propagation of superconducting correlations in the presence of the spin-orbit interaction and interface exchange field, in the diffusive regime which is relevant for our experiment. To describe the superconducting proximity effect, one can use the matrix Gorkov Green's function $\widehat{G}$ defined as:

$$\hat{G}(\vec{r}_1,\vec{r}_2,t_1,t_2)=\hat{\rho}_3\begin{bmatrix} G_{\psi_\uparrow\psi_\uparrow^\dagger} & G_{\psi_\uparrow\psi_\downarrow^\dagger} & G_{\psi_\uparrow\psi_\uparrow} & G_{\psi_\uparrow\psi_\downarrow} \\ G_{\psi_\downarrow\psi_\uparrow^\dagger} & G_{\psi_\downarrow\psi_\downarrow^\dagger} & G_{\psi_\downarrow\psi_\uparrow} & G_{\psi_\downarrow\psi_\downarrow} \\ G_{\psi_\uparrow^\dagger\psi_\uparrow^\dagger} & G_{\psi_\uparrow^\dagger\psi_\downarrow^\dagger} & G_{\psi_\uparrow^\dagger\psi_\uparrow} & G_{\psi_\uparrow^\dagger\psi_\downarrow} \\ G_{\psi_\downarrow^\dagger\psi_\uparrow^\dagger} & G_{\psi_\downarrow^\dagger\psi_\downarrow^\dagger} & G_{\psi_\downarrow^\dagger\psi_\uparrow} & G_{\psi_\downarrow^\dagger\psi_\downarrow} \end{bmatrix} \quad \text{(S3)}$$

The elements of the matrix Green's function (S3) are retarded Green's functions $G_{B,A}(t_1,t_2)=-i\theta(t_1-t_2)\langle\{\mathrm{B}(t_1),\mathrm{A}(t_2)\}\rangle$, where $\langle\ \ \rangle$ denotes the thermal average and $\{,\}$ is the anticommutator. For later use, we also define the commutator $[\,,]$. Due to the use of the field operators and their conjugates, the Green's function has a structure not only in the spin but also in the Nambu (electron/hole) space, and the matrix Green's function contains both the normal and 'anomalous' (superconducting) correlations. For later use we define spin Pauli matrices extended to the spinl⊗Nambu space, defined as $\hat{\sigma}_3=diag[1,-1,1,-1]$ and Nambu Pauli matrices such as $\hat{\rho}_3=diag[1,1,-1,-1]$ (the other Pauli matrices can be defined accordingly). For simplicity we use below $\hbar$=1. Note that we consider only the retarded Green's function because we study supercurrents which are equilibrium quantities. Since we consider a stationary problem, we make the Fourier time transform $\hat{G}(\vec{r}_1,\vec{r}_2,\varepsilon)=\int dte^{i\varepsilon(t_1-t_2)}\,\hat{G}(\vec{r}_1,\vec{r}_2,t_1,t_2)$. The Green's function $\hat{G}(\vec{r}_1,\vec{r}_2,\varepsilon)$ follows the Gorkov equations:

$$\left(\varepsilon\hat{\rho}_3+\frac{\hbar^2\nabla_{\vec{r}_1}^2}{2m}+\mu+i\frac{\alpha}{m}\left(\hat{\sigma}_2\frac{\partial}{\partial x_1}+\hat{\sigma}_1\hat{\rho}_3\frac{\partial}{\partial y_1}\right)+\hat{S}_{imp}\right)\hat{G}(\vec{r}_1,\vec{r}_2,\varepsilon)=\hat{1}\delta(\vec{r}_1,\vec{r}_2) \quad \text{(S4)}$$

$$\hat{G}(\vec{r}_1,\vec{r}_2,\varepsilon)\left(\varepsilon\hat{\rho}_3+\frac{\hbar^2\nabla_{\vec{r}_2}^2}{2m}+\mu-i\frac{\alpha}{m}\left(\hat{\sigma}_2\frac{\partial}{\partial x_2}+\hat{\sigma}_1\hat{\rho}_3\frac{\partial}{\partial y_2}\right)+\hat{S}_{imp}\right)=\hat{1}\delta(\vec{r}_1,\vec{r}_2) \quad \text{(S5)}$$

where $\hat{1}$ is the identity in the spin⊗Nambu space. These equations can be obtained by writing down the equations of motion for the field operators due to the Hamiltonian $\hat{H}_{N-Rashba}$. We have added a self-energy term $\hat{S}_{imp}$ which accounts for electronic scattering on the impurities of the material, along the standard approach of the quasiclassical theory of superconductivity.

In the limit where the electronic correlations evolve on a characteristic scale much larger than the Fermi wavelength, one can simplify the Gorkov description by making the quasiclassical approximation. We define the quasiclassical Eilenberger Green's function in

the mixed representation:

$$\hat{g}_\varepsilon(\vec{R},\vec{n}) = \frac{i}{\pi}\oint d\xi_p \int d^3\vec{r}\; e^{-i\vec{p}.\vec{r}}\check{G}(\vec{R}+\vec{r}/2\,;\vec{R}-\vec{r}/2\,,\varepsilon) \qquad \text{(S6)}$$

Due to the integration with respect to $\xi_p = p^2/2m$, $\hat{g}_\varepsilon$ depends only on the direction $\vec{n}$=$\vec{p}/p$ of $\vec{p}$. Making use of the gradient expansion technique and performing a semiclassical approximation, one obtains the following Eilenberger equation for the N-Rashba metal (*S4-S6*):

$$-iv_F\vec{n}.\vec{\tilde{\nabla}}_R\hat{g}_\varepsilon(\vec{R},\vec{n}) = \left[\varepsilon\hat{\rho}_3 + \hat{S}_{imp}, \hat{g}_\varepsilon(\vec{R},\vec{n})\right] + i\frac{\alpha}{2m}\left\{\left(\hat{\sigma}_2\frac{\partial}{\partial X} + \hat{\sigma}_1\hat{\rho}_3\frac{\partial}{\partial Y}\right), \hat{g}_\varepsilon(\vec{R},\vec{n})\right\} \qquad \text{(S7)}$$

with $\vec{\tilde{\nabla}}_R[\cdot] = \vec{\nabla}_R[\cdot] + i\alpha[\vec{x}\hat{\sigma}_2 + \vec{y}\hat{\sigma}_1\hat{\rho}_3,\ \cdot\,]$ the covariant gradient. The equation (S7) is the similar to what has been found by Tokatly and Bergeret for example in reference S2. The form of the spin orbit term is however slightly different and similar to references S5 and S6 (S6 being by the same authors as S2). We now define the isotropic Green's function:

$$\hat{\mathcal{G}}_\varepsilon(\vec{R}) = \int\frac{d\Omega}{4\pi}\hat{g}_\varepsilon(\vec{R},\vec{n}) \qquad \text{(S8)}$$

where $\int\frac{d\Omega}{4\pi}$ is an angular integration on the direction $\vec{n}$. This last Green's function is a relevant quantity when impurity scattering in the material is strong. The impurities yield an isotropization of the electronic correlations (*S4*), which leads to the Usadel equation:

$$D\vec{\tilde{\nabla}}_R.\hat{\mathcal{G}}_\varepsilon(\vec{R})\vec{\tilde{\nabla}}_R\hat{\mathcal{G}}_\varepsilon(\vec{R}) = \left[-i\varepsilon\hat{\rho}_3, \hat{\mathcal{G}}_\varepsilon(\vec{R})\right] + \frac{\alpha}{2m}\left\{\left(\hat{\sigma}_2\frac{\partial}{\partial X} + \hat{\sigma}_1\hat{\rho}_3\frac{\partial}{\partial Y}\right), \hat{\mathcal{G}}_\varepsilon(\vec{R})\right\} \qquad \text{(S9)}$$

where D is the diffusion constant in the material. The equation (S9) is also similar to the existing literature (*S2,S3*).

In the experimental setups considered here, and important aspect is the existence of a (ferri-)magnetic insulator (YIG) layer below the Rashba normal metal (Pt, Pd, W or Ta) which induces an interface exchange field. This overlapping geometry has already been considered in the reference (*S1*). The spin-active interface is shown to generate, through boundary conditions (*S4*), an exchange field in the direction of the magnetization (taken along the y axis in our case). This parameter is linked to the G_ϕ conductance (*S1,S4,S8*) of the YIG/normal metal interface

through. Importantly, this parameter can vary in *amplitude and sign* depending on the interface property (*S3*). We define it as γ_ϕ. Importantly, it has been shown that in the regime of small thickness of the normal part with respect to the superconducting coherence length, its effect can be incorporated in an effective 1D equation (*S1*). Thus, equation (S9) becomes:

$$D\vec{\vec{\nabla}}_R.\hat{\mathcal{G}}_\varepsilon(\vec{R})\vec{\vec{\nabla}}_R\hat{\mathcal{G}}_\varepsilon(\vec{R}) = \left[-i\varepsilon\hat{\rho}_3 + i\gamma_\phi\hat{\rho}_3\hat{\sigma}_2, \hat{\mathcal{G}}_\varepsilon(\vec{R})\right] + \frac{\alpha}{2m}\left\{\left(\hat{\sigma}_2\frac{\partial}{\partial X} + \hat{\sigma}_1\hat{\rho}_3\frac{\partial}{\partial Y}\right), \hat{\mathcal{G}}_\varepsilon(\vec{R})\right\}$$

(S10)

It is the combination of γ_ϕ and α which accounts for the φ_0 −Josephson junction behavior. We now calculate the supercurrent arising from the proximity effect described by (S10). We assume that the two superconducting electrodes are separated by a length L along the x axis and have a superconducting phase of $\pm\varphi/2$.

The expression of the average current flowing through the normal metal junction of conductivity σ_N and surface S can be expressed as:

$$I = \frac{-i\pi}{4e} k_B T\, S\, \sigma_N \sum_{\omega_n} Tr\left[\hat{\rho}_3\hat{\mathcal{G}}_{\omega_n}(\vec{R})\vec{\vec{\nabla}}_R\hat{\mathcal{G}}_{\omega_n}(\vec{R})\right] \quad \text{(S11)}$$

Above, we use Usadel Green's functions which depend on the Matsubara frequencies $\omega_n = (2n+1)\pi k_B T$ instead of the real energy ε because it will simplify the calculation of the current. One can formally relate the Usadel equations in the two pictures by making the substitution $-i\varepsilon \rightarrow \omega_n$. We consider the case of weak proximity effect where superconducting correlation functions can be introduced perturbatively. This regime is proper to obtain simple formulae but does not account for higher harmonics in the current phase relationship. We leave the discussion on the higher order harmonics to subsequent studies of the non-linear problem and focus here on the effect of γ_ϕ and α on the φ_0-Josephson junction behavior which persists even in the linearized regime. The Green's function of the problem can be sought for with the form:

$$\hat{\mathcal{G}}_\varepsilon(\vec{R}) = \begin{bmatrix} sgn\,(\omega_n) & 0 & sgn\,(\omega_n)\mathcal{F}_t(x) & -\mathcal{F}_s(x) \\ 0 & sgn\,(\omega_n) & \mathcal{F}_s(x) & sgn\,(\omega_n)\mathcal{F}_t(x) \\ sgn\,(\omega_n)\mathcal{F}_t(x)^* & -\mathcal{F}_s(x)^* & -sgn\,(\omega_n) & 0 \\ \mathcal{F}_s(x)^* & sgn\,(\omega_n)\mathcal{F}_t(x)^* & 0 & -sgn\,(\omega_n) \end{bmatrix}$$

where $\mathcal{F}_s(x)\ and\ \mathcal{F}_t(x)$ are respectively the singlet, triplet components. The Usadel equation (S10) can be simplified into two coupled equations:

$$\hbar D\partial_x^2\mathcal{F}_s - 2|\omega_n|\,\mathcal{F}_s + 2\gamma_\phi\mathcal{F}_t - i\frac{\hbar\alpha}{m}\partial_x\mathcal{F}_t = 0 \qquad \text{(S12a)}$$

$$\hbar D\partial_x^2\mathcal{F}_t - \left[2|\omega_n| + 4\frac{D\alpha^2}{\hbar}\right]\mathcal{F}_t - 2\gamma_\phi\mathcal{F}_s + i\frac{\hbar\alpha}{m}\partial_x\mathcal{F}_s = 0 \qquad \text{(S12b)}$$

As expected, the above equations are similar to those of a ferromagnetic Josephson junction with Rashba spin-orbit interaction and an exchange field $\Delta E_{ex} = -\gamma_\phi$. Since we are interested in the high temperature regime for which $2|\omega_n| \gg 4\frac{D\alpha^2}{\hbar}$, we neglect the $4\frac{D\alpha^2}{\hbar}$ term which is in fact not relevant for the physics discussed here. The proper boundary conditions for the overlapping geometry have also been derived in S1. In our case, we assume that the interface transparency between the superconductor and the N-Rashba material is weak as found in S9. The boundary conditions for $\mathcal{F}_s(x), \mathcal{F}_t(x)$ read[S1]:

$$\mathcal{F}_s(x=0) = \gamma_T\frac{\Delta e^{+i\varphi/2}}{\sqrt{\Delta^2+{\omega_n}^2}} \qquad \text{(S13a)}$$

$$\mathcal{F}_s(x=L) = \gamma_T\frac{\Delta e^{-i\varphi/2}}{\sqrt{\Delta^2+{\omega_n}^2}} \qquad \text{(S13b)}$$

$$\mathcal{F}_t(x=0) = 0 \qquad \text{(S13c)}$$

$$\mathcal{F}_t(x=L) = 0 \qquad \text{(S13d)}$$

which relate $\mathcal{F}_s(x)$ and $\mathcal{F}_t(x)$ to the values of the superconducting correlations below the superconducting electrodes (Δ is the superconducting gap in the superconducting electrodes).

We take the simplified limit here of an $\mathcal{F}_s(x)$ function proportional to the BCS F-function which is valid if the spin orbit and the interface exchange field have perturbative effects (*S1*). The parameter γ_T accounts for the transparency of the tunnel barrier. The set of

equations (S12a, S12b) may be solved by introducing the vector

$F = \quad {}^{t}(\partial_x \mathcal{F}_s(x), \partial_x \mathcal{F}_t(x), \mathcal{F}_s(x), \mathcal{F}_t(x)).$

In the linear regime and in the long junction regime where the decay length of the correlation functions is small with respect to L, we may write the solutions of (S12a) and (S12b) satisfying the boundary conditions as:

$$\begin{bmatrix}\mathcal{F}_s \\ \mathcal{F}_t\end{bmatrix} = \gamma_T \frac{\Delta}{\sqrt{\Delta^2+\omega_n{}^2}} \left\{ \begin{bmatrix}1 \\ \text{-i}\end{bmatrix} \left[e^{\lambda_1 x} e^{i\varphi/2} + e^{\lambda_3(x-L)} e^{-i\varphi/2} \right] + \begin{bmatrix}1 \\ i\end{bmatrix} \left[e^{\lambda_2 x} e^{i\varphi/2} + e^{\lambda_4(x-L)} e^{-i\varphi/2} \right] \right\}$$

(S14)

The wavevectors $\lambda_1, \lambda_2, \lambda_3, \lambda_4$ are the eigenvalues of the matrix governing the equation of motion for the vector F. We keep the theory to lowest order in γ_ϕ *and* $\tilde{\alpha}$ which allows to capture the essence of the physics involved. We can write: $\lambda_1 = \tilde{\alpha} - k_{\omega_n} - i\tilde{\gamma}_\phi, \lambda_2 = -\tilde{\alpha} - k_{\omega_n} + i\tilde{\gamma}_\phi, \lambda_3 = \tilde{\alpha} + k_{\omega_n} + i\tilde{\gamma}_\phi, \lambda_4 = -\tilde{\alpha} + k_{\omega_n} - i\tilde{\gamma}_\phi$ with $k_{\omega_n} = \sqrt{2|\omega_n|/\hbar D}$, $\tilde{\alpha} = \frac{\alpha}{2mD}$ and $\tilde{\gamma}_\phi = \frac{\gamma_\phi}{\sqrt{2\hbar D|\omega_n|}}$. Using formula (S11), we get the following expression for the supercurrent:

$$I = \frac{2\pi}{e} k_B T\, S\, \sigma_N \sum_{\omega_n} \gamma_T^2 \frac{\Delta^2}{\omega_n{}^2+\Delta^2} k_{\omega_n} e^{-k_{\omega_n} L} \big[-Cos(\varphi) Sin(\tilde{\gamma}_\phi L) Sinh(\tilde{\alpha} L) + Sin(\varphi) Cos(\tilde{\gamma}_\phi L) Cosh(\tilde{\alpha} L) \big] \quad \text{(S15)}$$

This is the φ_0-junction's current phase relation (CPR). To lowest order in $\tilde{\gamma}_\phi$ *and* $\tilde{\alpha}$, we expand the above formula and get:

$$I \approx I_C \sin\big(\varphi - \tilde{\alpha}\tilde{\gamma}_\phi L^2\big) \qquad \text{(S16)}$$

with $I_C = \frac{2\pi}{e} k_B T\, S\, \sigma_N \sum_{\omega_n} \gamma_T^2 \frac{\Delta^2}{\omega_n{}^2+\Delta^2} k_{\omega_n} e^{-k_{\omega_n} L}$. Thus, the junction is a φ_0- Josephson junction with $\varphi_0 = -\tilde{\alpha}\tilde{\gamma}_\phi L^2 = -\frac{\alpha\gamma_\phi}{\sqrt{8m^2D^3\hbar|\omega_n|}} L^2 \approx -\frac{\alpha\gamma_\phi}{\sqrt{8m^2D^3\hbar|\pi k_B T|}} L^2$, taking the high temperature limit for the Matsubara frequencies. Combined with the higher order harmonics which are naturally present in all SNS junctions, it provides a microscopic explanation of the diode effect. Interestingly, the $\tilde{\gamma}_\phi$ parameter is linked to the G_ϕ conductance (*S1,S4,S9*) of

the YIG/normal metal interface and can vary in *amplitude and sign* depending on the interface (*S3*). As summarized in Fig. 5, the φ_0 data approximately scale linearly with the $Q_{\mu_0 H=0}$ data, which is well consistent with our theoretical prediction and establishes a direct correlation between these quantities. This provides a simple explanation both for the sign and magnitude of φ_0 observed in the data and the non-volatile nature of the anomalous phase shift.

Quantitative estimates of loop inductance and flux offset.

Essentially, the screening parameter $\beta_m = \frac{2\Phi_s}{\Phi_0}$ (*34*), where $\Phi_s = LI_c$ is the screening flux generated by the circulating supercurrent in the SQUID loop, with L the geometric inductance, I_c the critical current of each JJ and $\Phi_0 = 2.07\times10^{-15}$ T·m^2 the flux quantum, provides a quantitative criterion for determining whether magnetic hysteresis can occur in the SQUID.

For our SQUID ($L\approx$ 5 pH (*S20*) I_c = 70–105 μA), $\beta_m = \frac{2LI_c}{\Phi_0}$ = 0.4–0.7 $\lesssim$ 1. This allows us to rule out trapped flux as a source of residual phase shifts.

REFERENCES

S1. A. Cottet. Inducing odd-frequency triplet superconducting correlations in a normal metal. *Phys. Rev. Lett.* **107**, 177001 (2011).

S2. F. S. Bergeret, I. V. Tokatly. Theory of diffusive φ_0 Josephson junctions in the presence of spin–orbit coupling. *Europhys. Lett.* **110**, 57005 (2015).

S3. A. Cottet, T. Kontos, et al. Semiconductor Sci. Technol. **21**, S78 (2006).

S4. M. Eschrig, A. Cottet, W. Belzig, J. Linder. General boundary conditions for quasiclassical theory of superconductivity in the diffusive limit: application to strongly spin-polarized systems. *New J. Phys.* **17**, 083037 (2015).

S5. A. Zyuzin, M. Alidoust, D. Loss. Josephson junction through a disordered topological

insulator with helical magnetization. *Phys. Rev. B* **93**, 214502 (2016).

S6. I. V. Tokatly, F. S. Bergeret. Spin-orbit coupling as a source of long-range triplet proximity effect in superconductor-ferromagnet hybrid structures. *Phys. Rev. B* **89**, 134517 (2014).

S7. The spin rotation effect at a spin-active interface should be described with appropriate boundary conditions for the Usadel Green's functions. Such boundary conditions have already been derived for the SC/FM spin-active interfaces, see for instance M. Eschrig, A. Cottet, W. Belzig, J. Linder, *New J. Phys.* **17** 083037 (2015) and A. Cottet, D. Huertas-Hernando, W. Belzig, and Y. V. Nazarov, *Phys. Rev. B* **80**, 184511 (2009). These boundary conditions explicitly take into account the interfacial spin-precession effect and the interfacial conversion of the spin-unpolarised singlets ($S = 0$) through the spin-zero ($S = 1, m_s = 0$) to the spin-polarised triplets ($S = 1$, $m_s = \pm 1$).

S8. A. Cottet, D. Huertas-Hernando, W. Belzig, Y. V. Nazarov. Spin-dependent boundary conditions for quasiclassical theory of superconductivity in diffusive systems. *Phys. Rev. B* **80**, 184511 (2009).

S9. T. Kontos, M. Aprili, J. Lesueur, X. Grison, L. Dumoulin. Superconducting proximity effect at the paramagnetic-ferromagnetic transition. *Phys. Rev. Lett.* **93**, 137001 (2004).

S10. N. Vlietstra, J. Shan, G. Wolfs, A. Bollinger, A. Gupta, B. Koopmans, Exchange magnetic field torques in YIG/Pt bilayers observed by the spin-Hall magnetoresistance. *Appl. Phys. Lett.* 103, 032401 (2013).

S11. Y.-T. Chen, S. Takahashi, H. Nakayama, T. Ueda, M. Althammer, Theory of spin Hall magnetoresistance. *Phys. Rev. B* **87**, 144411 (2013).

S12. X.-P. Zhang, F. S. Bergeret, V. N. Golovach, Theory of spin Hall magnetoresistance from a microscopic perspective. *Nano Lett.* **19**, 6330–6337 (2019).

S13. H. L. Wang, C. H. Du, Y. Pu, R. Adur, P. C. Hammel, F. Y. Yang, Scaling of spin Hall angle in 3d, 4d, and 5d metals from $Y_3Fe_5O_{12}$/metal spin pumping. *Phys. Rev. Lett.* **112**, 197201

(2014).

S14. L. Ma, Y.-T. Chen, J. Shan, L. Du, L. Sheng, R. Yang, Q. Niu, K. Xia, J. Shi, Spin diffusion length and spin Hall angle in $Pd_{1-x}Pt_x$/YIG heterostructures: Examination of spin relaxation mechanism. *Phys. Rev. B* **98**, 224424 (2018).

S15. K.-R. Jeon, J.-C. Jeon, X. Zhou, A. Migliorini, J. Yoon, S. S. P. Parkin, Giant transition-state quasiparticle spin-Hall effect in an exchange-spin-split superconductor detected by nonlocal magnon spin transport. *ACS Nano* **14**, 15874–15883 (2020).

S16. K.-R. Jeon, C.-Y. You, J. Shin, S.-W. Lee, D.-J. Kim, J.-H. Kang, H. Kim, J. Kang, J. Won, K. Cho, A. Chakraborty, J.-C. Jeon, J. Yoon, H. Han, J.-K. Kim, S. S. P. Parkin, Role of two-dimensional Ising superconductivity in the nonequilibrium quasiparticle spin-to-charge conversion efficiency. *ACS Nano* **15**, 16819–16827 (2021).

S17. L. J. Cornelissen, J. Shan, J. Y. Cai, X. Lu, X. Gong, B. Jin, R. Duine, B. J. van Wees, Magnon spin transport driven by the magnon chemical potential in a magnetic insulator. *Phys. Rev. B* **94**, 014412 (2016).

S18. S. A. Bender, R. A. Duine, Y. Tserkovnyak, Electronic pumping of quasi-equilibrium Bose-Einstein-condensed magnons. *Phys. Rev. Lett.* **108**, 246601 (2012).

S19. J. Shan, L. Chen, Q. Zhang, Z. Wang, L. Wu, P. Huang, Influence of yttrium iron garnet thickness and heater opacity on the nonlocal transport of electrically and thermally excited magnons. *Phys. Rev. B* **94**, 174437 (2016).

S20. I. Sochnikov

S20. D. Davino, B. Kaliszky, dc SQUID design with femtotesla sensitivity for quantum-ready readouts. *Phys. Rev. Appl.* **14**, 014020 (2020).

FIGURE CAPTIONS

Fig. S1. X-ray structural analysis of Josephson barriers. Theta-2theta scans of (**A**) Pt, (**B**)

W, (**C**) Ta, and (**D**) Pd thin films grown on the YIG/GGG (111) substrates. Pt (111), α-Ta (110), α-W (110), and Pd (111) films are oriented on the substrates, respectively. X-ray reflectivity (XRR) measurements of (**E**) Pt, (**F**) W, (**G**) Ta, and (**H**) Pd thin films on YIG/GGG (111) substrates. Each thickness of the thin films is determined from the oscillation period of the fringe peaks. Note that the YIG film is not reflected owing to its large thickness of ~200 nm. The teta-2theta scans and XRR measurements were carried out using a Bruker D8 Discovery X-ray diffractometer with Cu-Kα radiation at 40 kV and 40 mA.

Fig. S2. Comparison of measurement configurations. In our SQUID geometry, the maximum $\varphi_{tot}(= \varphi_0^1 \sin\left(\frac{\pi}{4}\right) + \varphi_0^2 \sin\left(\frac{\pi}{4}\right) \approx 2\varphi_0 \sin\left(\frac{\pi}{4}\right)$, where the pre-factor 2 accounts for the doubled anomalous phases acquired by two symmetric JJs around the SQUID loop and the post-factor $\sin\left(\frac{\pi}{4}\right)$ reflects the magneto-chiral effect), can be obtained by setting the directions of I_s^1 and I_s^2 across two constituent JJs to be inclined with respect to the remanent-state $M_{\varphi_0-barrier}$ (**A**). On the other hand, the non-zero $Q_{\mu_0 H = 0}$ [half that of a single φ_0-junction in Fig. 2] can be detected by setting the orientations of I_s^1 and I_s^2 to be either perpendicular and parallel to the remanent-state $M_{\varphi_0-barrier}$, or vice versa (**B**). Note that in the first measurement configuration, the time-averaged voltage $V_\pm$ is measured as a function of perpendicular magnetic field $\mu_0 H_\perp$ for the d.c. current *I*-biased SQUID (Fig. 4). In the second measurement configuration, the current-voltage *I*-*V* curves are detected at zero external field $\mu_0 H = 0$ (Fig. S5). In this case, due to the magneto-chirality, either I_s^1 or I_s^2 ($\perp M_{\varphi_0-barrier}$) can contribute to the resulting diode effect in $I_s^{tot} = I_s^1 + I_s^2$. For both configurations, the remanent-state $M_{\varphi_0-barrier}$ is preconfigured by applying an IP magnetic field $\mu_0 H_\parallel$ = 30 mT and then returning it to zero.

Fig. S3. Estimation of exchange spin-splitting from anomalous Hall effect measurements.

(**A**) Schematic illustration of Hall-bar device and measurement geometry (*5,S10*). (**B**) Anomalous Hall resistivity ρ_{AH} as a function of perpendicular magnetic field $\mu_0 H_\perp$ for the Pt(4 nm) Hall-bar device, taken at the fixed temperature T = 2 K. Here $\rho_{AH} = R_{AH} t_{Pt}$, R_{AH} is the anomalous Hall resistance and t_{Pt} is the Pt thickness. The top inset shows a schematic of the Pt Hall-bar device and measurement geometry used. The bottom inset exhibits the T evolution of the longitudinal Pt resistivity ρ_{yy}. (**C**), (**D**), and (**E**) Data equivalent to (B) but for the Ta(4 nm), W(4 nm) and Pd(4 nm) Hall-bar devices, respectively, in (C), (D), and (E). (**F**) According to a spin-Hall AHE theory (*S11,S12*), the AH resistivity is given by $\Delta\rho_{AH} = -\frac{2(l_{sd}\theta_{SH})^2 \rho}{t} \mathrm{Im} \frac{G_{\uparrow\downarrow} \tanh^2\left(\frac{t}{2l_{sd}}\right)}{\frac{1}{\rho} + 2l_{sd} G_{\uparrow\downarrow} \coth\left(\frac{t}{l_{sd}}\right)} \approx -\frac{2(l_{sd}\theta_{SH})^2}{t} \frac{G_i \tanh^2\left(\frac{t}{2l_{sd}}\right)}{\left(\frac{1}{\rho} + 2l_{sd} G_r \coth\left(\frac{t}{l_{sd}}\right)\right)^2}$, where $G_{\uparrow\downarrow} = G_r + iG_i$ is the spin-mixing conductance at the interface between the proximity layer and YIG, and in the limit that the real part is much larger than the imaginary one, $G_r \gg G_i$. l_{sd}, θ_{SH}, ρ [insets of (B), (C), (D), and (E)], and t (= 4 nm) are the spin-diffusion length, spin-Hall angle, electric resistivity, and thickness of the proximity layer, respectively. Using $\Delta E_{ex} = \frac{|G_i|}{\left(\frac{e^2}{\hbar}\right) g_F t}$ (*S12*) and the estimated values (*S12- S14*) from similar structures, we estimate ΔE_{ex} to first order for each proximity layer in (F). Here $\hbar$ is the reduced Planck's constant and g_F is the density-of-states of the proximity layer at the Fermi level.

Fig. S4. Characterization of spin-orbit coupling from non-local thermal-magnon transport measurements. (**A**) Schematic illustration of non-local magnon device (MD) and measurement scheme (*S15,S16*). (**B**) Thermally driven nonlocal voltage ΔV_{nl}^{th} as a function of in-plane field angle α for the non-local MD with a pair of proximity-layer electrodes, taken at I = |0.5| mA at T = 2 K. The black solid line is a sin(α) fit. (**C**), (**D**), and (**E**) Data equivalent to (B) but for the non-local MDs with Ta(4 nm), W(4 nm), and Pd(4 nm) electrodes, respectively, in (C), (D), and (E). (**F**) Theoretically [*S17-S19*], one can express $\Delta V_{nl}^{th} =$

$\theta_{SH} j_s \left(\frac{l_{sd}}{t}\right) \tanh\left(\frac{t}{2l_{sd}}\right)\left(\frac{\mathrm{e}}{\hbar}\right) l$, where j_s and l (≈ 50 μm) are the thermally-driven magnon-spin current density at the interface and the length of the detector electrode, respectively. Using the above formula and the estimated values (*S12-S14*) from similar structures, we calculate the normalized θ_{SH} by the Pt's spin-Hall angle for each proximity layer in (F). In this calculation, j_s is assumed to be constant for simplicity.

Fig. S5. Zero-field polarity-switchable supercurrent diode effect detected in the SQUID consisting of symmetric JJs. (**A**) Zero-field current-voltage *I-V* curves of the Pt JJ-based SQUID for two different remanent-state $M_{\varphi_0-barrier}$ ($\perp I_s^1$ // I_s^2), taken at $T = 2$ K. Here I is normalized by $2I_c^{avg}$ = 145−210 μA and I_c^{avg} is the averaged critical current of each JJ. Note that in this configuration, I_s^1 ($\perp M_{\varphi_0-barrier}$) can only contribute to the resulting diode effect due to the magneto-chirality (Fig. 3F), halving the magnitude of $Q_{\mu_0 H\,=\,0}$ compared with data in Fig. 2A. In the yellow (cyan) shaded regime, the Josephson supercurrent flows only in the positive (negative) direction, as indicated by the diode symbols. (**B**), (**C**), and (**D**) Data equivalent to (A) but for the Ta JJ-based, W JJ-based and Pd JJ-based SQUIDs, respectively, in (B), (C), and (D).

Table S1. Comparison between exchange–spin-split (this work) and Zeeman–spin-split (field-driven) φ_0 junctions.

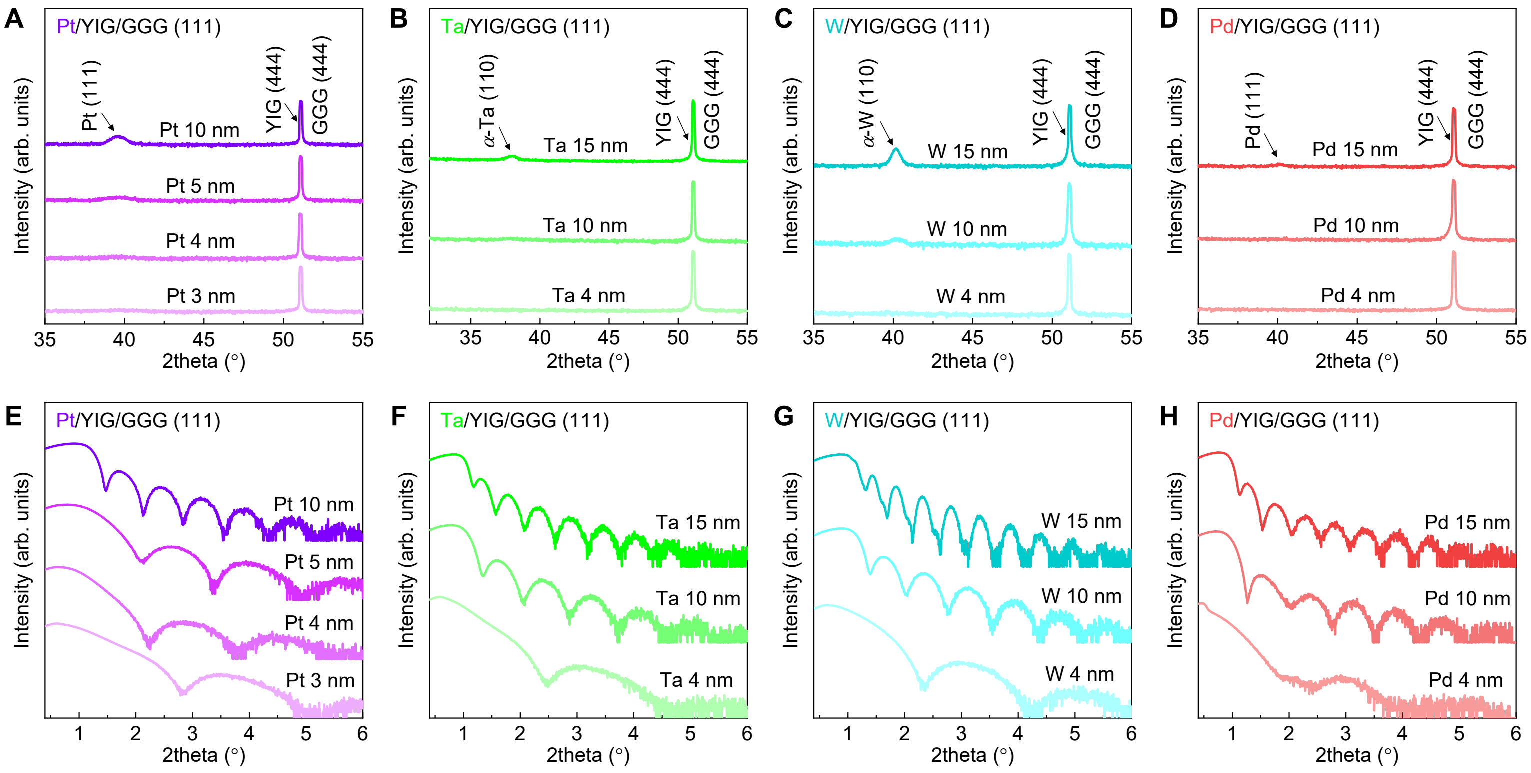

A
Pt/YIG/GGG (111)
Pt (111)
YIG (444)
GGG (444)
Pt 10 nm
Pt 5 nm
Pt 4 nm
Pt 3 nm
B
Ta/YIG/GGG (111)
α-Ta (110)
Ta 15 nm
Ta 10 nm
Ta 4 nm
C
W/YIG/GGG (111)
α-W (110)
W 15 nm
W 10 nm
W 4 nm
D
Pd/YIG/GGG (111)
Pd (111)
Pd 15 nm
Pd 10 nm
Pd 4 nm
E
F
G
H
Intensity (arb. units)
2theta (°)

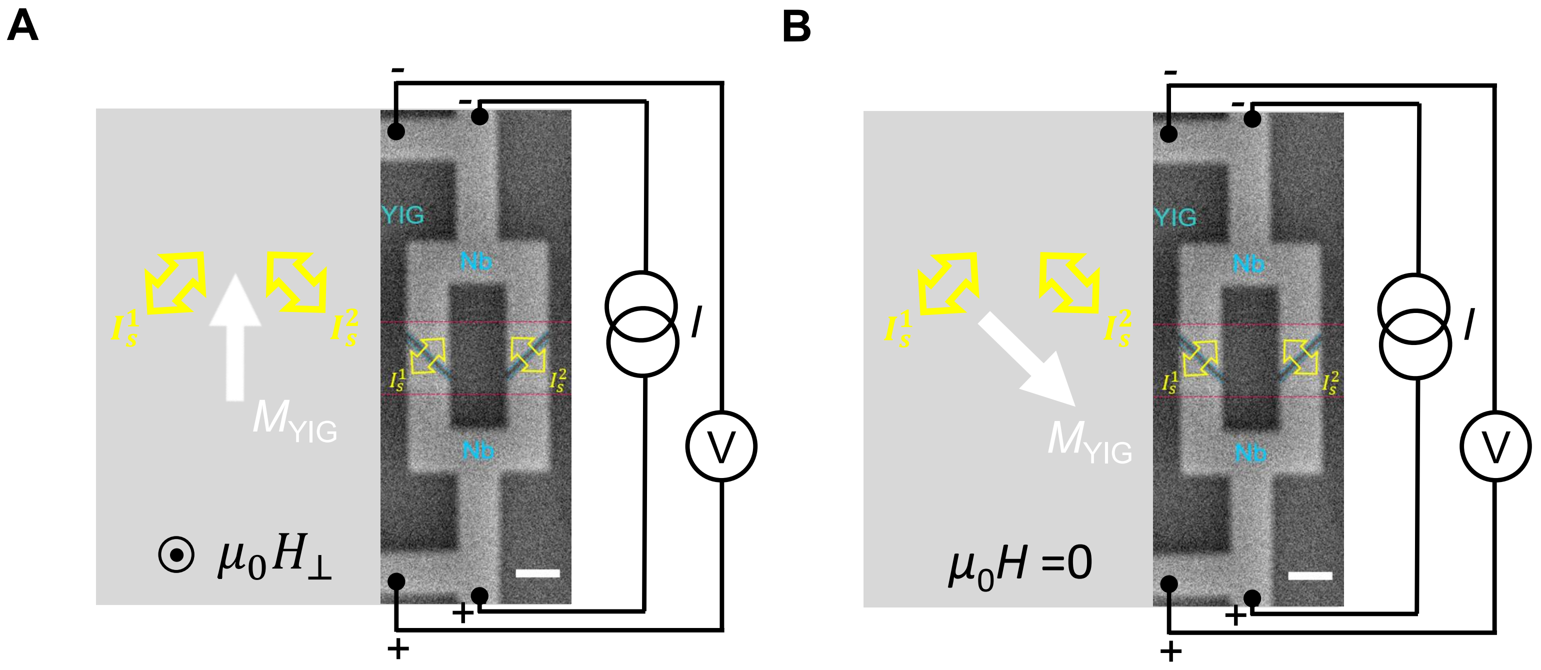
A
B
YIG
Nb
Nb
I_s^1
I_s^2
M_{YIG}
$\mu_0 H_\perp$
$\mu_0 H = 0$
I
V
-
+

A

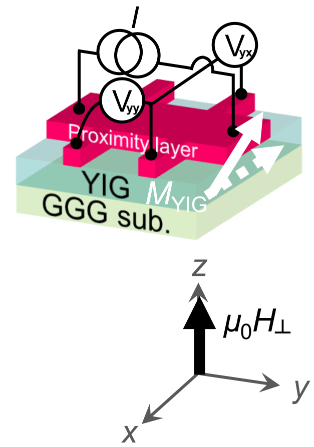

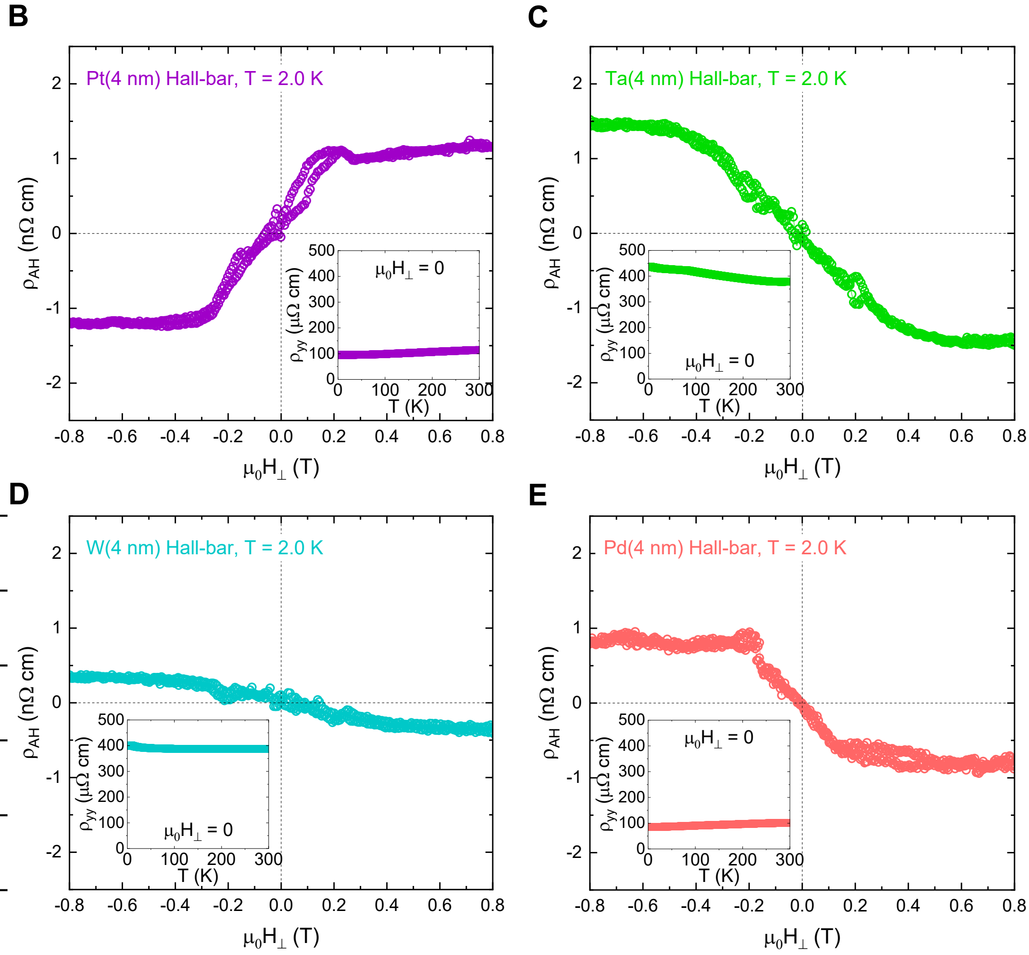

F

	G_r (Ω^{-1} m^{-2})	l_{sd} (nm)	θ_{SH}	G_i (Ω^{-1} m^{-2})	g_F (m^{-3}eV^{-1})	ΔE_{ex} (meV)
Pt/ YIG	4.4 $\times 10^{14}$	1.2	+ 0.08	-1.1 $\times 10^{13}$	3-4 $\times 10^{28}$	1.1
Ta/ YIG	5.4 $\times 10^{14}$	2	- 0.07	+0.8 $\times 10^{13}$	3-4 $\times 10^{28}$	0.8
W/ YIG	4.5 $\times 10^{14}$	2	- 0.14	+0.3 $\times 10^{13}$	3-4 $\times 10^{28}$	0.3
Pd/ YIG	4 $\times 10^{14}$	5	+ 0.05	-1.0 $\times 10^{13}$	3-4 $\times 10^{28}$	1.0

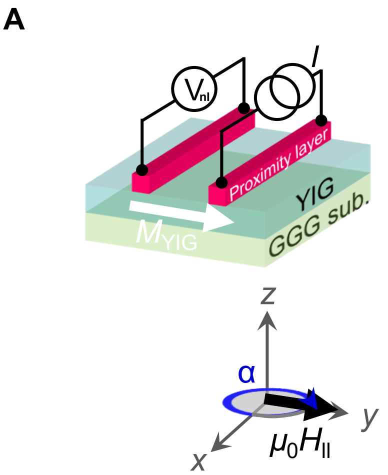

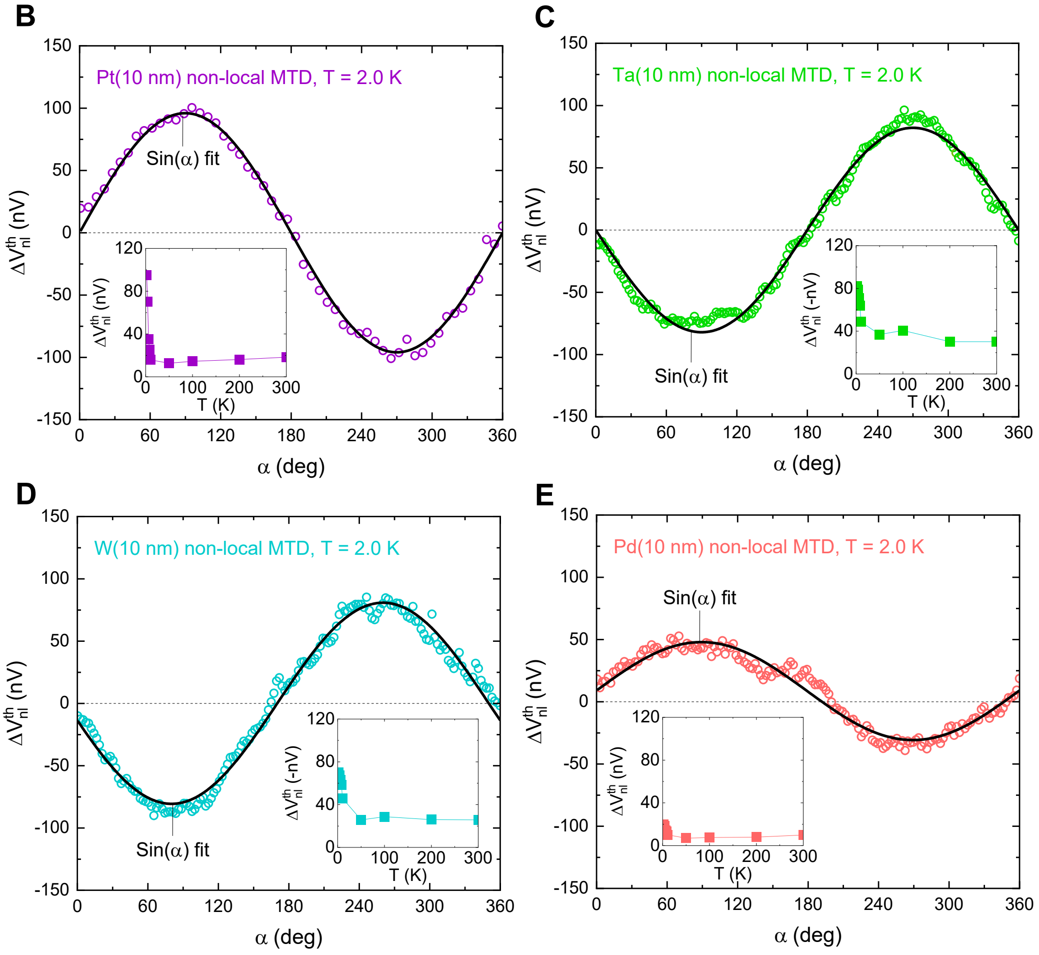

F

	Normalized θ_{SH}
Pt/YIG/Pt	+1.0
Ta/YIG/Ta	-0.5
W/YIG/W	-0.5
Pd/YIG/Pd	+0.1

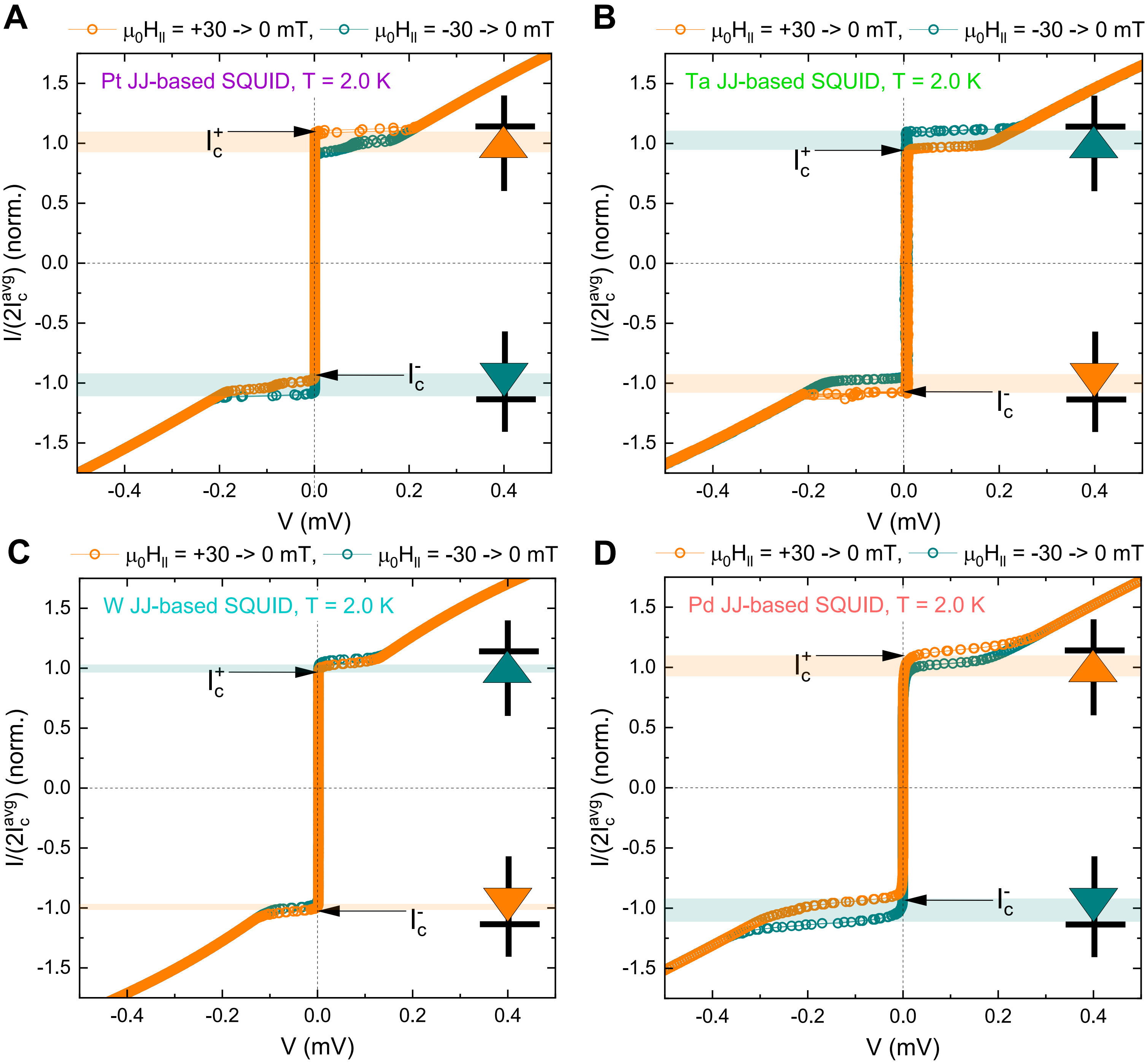
A
B
C
D
$\mu_0H_{||}$ = +30 -> 0 mT, $\mu_0H_{||}$ = -30 -> 0 mT
Pt JJ-based SQUID, T = 2.0 K
Ta JJ-based SQUID, T = 2.0 K
W JJ-based SQUID, T = 2.0 K
Pd JJ-based SQUID, T = 2.0 K
I_c^+
I_c^-
$I/(2I_c^{avg})$ (norm.)
V (mV)

**Property**	**Exchange–spin-split φ_0 junction (this work)**	**Zeeman–spin-split φ_0 junction (field-driven)**
Mechanism of time-reversal symmetry breaking	Broken by the ferromagnetic exchange interaction at the interface(s).	Broken by an externally applied magnetic field penetrating the junction.
Control method	Non-volatile control through material choice (ferromagnet type, thickness) and interfacial engineering.	Field-driven; requires continuous application or sweeping of an external magnetic field.
Selective / individual controllability	Possible: different metals or interfaces can be engineered to give distinct ΔE_{ex} contributions, enabling device-level selectivity.	Limited: magnetic field typically acts globally on all junctions and is not easily confined to specific devices.
Memory functionality	Non-volatile: exchange field remains without power or external stimuli.	Volatile: the Zeeman-induced φ_0 shift vanishes when the field is removed.
Achievable spin-splitting field	Exchange fields typically 1–10 T at the interface, depending on interface quality and material choice.	Zeeman fields generally $\leq$1−10 T, limited by the upper critical field of the superconducting electrodes.
Device scalability	High: no external field lines required; suitable for dense superconducting circuits.	Lower: magnetic field generation and shielding constraints hinder scalability.
Primary limitation	Quantitative extraction of the interfacial Rashba/exchange parameters remains challenging; depends strongly on interface quality.	Requires strong external fields, which can degrade superconductivity or produce crosstalk.
